# Endogenous Opsin 3 (OPN3) Protein Expression in the Adult Brain Using a Novel OPN3-mCherry Knock-In Mouse Model

**DOI:** 10.1523/ENEURO.0107-20.2020

**Published:** 2020-09-02

**Authors:** Lauren E. Olinski, Ayumi C. Tsuda, Julie A. Kauer, Elena Oancea

**Affiliations:** 1 Department of Molecular Biology, Cell Biology, and Biochemistry, Brown University, Providence, RI 02912; 2 Department of Molecular Pharmacology and Physiology, Brown University, Providence, RI 02912; 3 Department of Psychiatry, Stanford University, Stanford, CA 94305

**Keywords:** CRISPR/Cas9, encephalopsin, mouse, OPN3, opsin

## Abstract

The opsins have been studied extensively for their functions in visual phototransduction; however, the mechanisms underlying extraocular opsin signaling remain poorly understood. The first mammalian extraocular opsin to be discovered, opsin 3 (OPN3), was found in the brain more than two decades ago, yet its function remains unknown. A significant hindrance to studying OPN3 has been a lack of specific antibodies against mammalian OPN3, resulting in an incomplete understanding of its expression in the brain. Although *Opn3* promoter-driven reporter mice have been generated to examine general OPN3 localization, they lack the regulated expression of the endogenous protein and the ability to study its subcellular localization. To circumvent these issues, we have created a knock-in OPN3-mCherry mouse model in which the fusion protein OPN3-mCherry is expressed under the endogenous *Opn3* promoter. Viable and fertile homozygotes for the *OPN3-mCherry* allele were used to create an extensive map of OPN3-mCherry expression across the adult mouse brain. OPN3-mCherry was readily visualized in distinct layers of the cerebral cortex (CTX), the hippocampal formation (HCF), distinct nuclei of the thalamus, as well as many other regions in both neuronal and non-neuronal cells. Our mouse model offers a new platform to investigate the function of OPN3 in the brain.

## Visual Abstract

**Figure:**
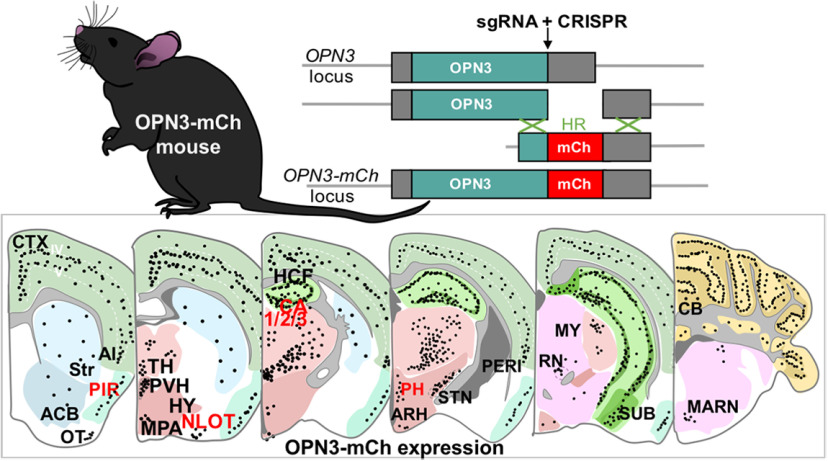

## Significance Statement

Before the current report, there had been a significant lack of encephalic opsin 3 (OPN3) protein characterization, likely driven by the absence of murine OPN3 antibodies. This study describes a novel OPN3-mCherry knock-in mouse that we used to analyze endogenous OPN3 protein expression across the brain. We have uncovered new aspects of OPN3: localization to previously undocumented brain subregions, expression in GABAergic neurons and non-neuronal cells, and punctate subcellular localization in the soma. Our OPN3 expression map is an invaluable step toward discovering its elusive encephalic functions. The OPN3-mCherry mouse will facilitate investigation of OPN3 not only in the brain, but also across the entire organism, a useful feature as OPN3 is emerging as a possible mediator of phototransduction outside the brain.

## Introduction

Opsins form a family of light-sensitive G-protein-coupled receptors (GPCRs) that function in a multitude of phototransduction mechanisms (Terakita, 2005). We depend on light to see, and as such, opsins have consistently been the research focus behind visual phototransduction, with retina-residing opsins being the most frequently investigated. Only recently have mammalian extraretinal opsins and their non-image forming functions become a major field of research.

The first opsin to be identified as having expression outside of the eye was opsin 3 (OPN3; Blackshaw and Snyder, 1999). OPN3 was initially named *encephalopsin* for its abundant expression in the mouse brain (Blackshaw and Snyder, 1999); it was later termed *panopsin* after discovery of its widespread expression in peripheral organs in mammals (Halford et al., 2001). Despite its discovery more than two decades ago, OPN3 remains one of the least characterized opsins and its expression profile and function are unknown in many of the tissues where it is expressed. The studies published thus far have focused primarily on OPN3 expression or function in the skin (for review, see Olinski et al., 2020), lungs (White et al., 2008; Barreto Ortiz et al., 2018; Yim et al., 2019), colon (Yoshimoto et al., 2018), liver (Jiao et al., 2012), and adipocytes (Nayak et al., 2020; Sato et al., 2020), but OPN3 has yet to gain attention in the brain, where it was first discovered.

Initially, OPN3 mRNA was identified by *in situ* hybridization in several murine brain areas including: cerebral cortex (CTX), striatum, preoptic areas, lateral thalamus, paraventricular nucleus of the hypothalamus (PVH), spinal cord, and cerebellar Purkinje cells (Blackshaw and Snyder, 1999). It was only in 2012 that OPN3 protein expression was examined in the adult mouse brain through immunofluorescence and immunoblotting for endogenous OPN3 (Nissilä et al., 2012). However, this study was unable to specifically dissect OPN3 expression across the entire brain, and some results were in direct opposition to previous reports; for example, OPN3 mRNA was highly expressed in the testes (Blackshaw and Snyder, 1999; Halford et al., 2001), but no expression was found there by Nissilä et al. (2012). The promiscuity of many OPN3 antibodies may have been the cause of this discrepancy. Years later, a report in 2018 characterized OPN3 protein expression in giant choline acetyltransferase (ChAT)-expressing and small non-ChAT-expressing neurons of the monkey striatum (El Massri et al., 2018), but this study was again limited by the specificity of commercially available OPN3 antibodies.

To circumvent the dependence on antibodies for the complete characterization of OPN3 across tissues, we have used CRISPR/Cas9 gene editing to create a knock-in mouse model which expresses mCherry (mCh)-tagged OPN3 under the endogenous *Opn3* promoter. The advantage of this reporter mouse over other models is that OPN3-mCh fusion protein is expressed under the native promoter, preserving endogenous levels of OPN3 with native expression across development. This is fundamental for furthering OPN3 characterization as OPN3 has been shown to be developmentally regulated, at least in the brain (Blackshaw and Snyder, 1999; Lein et al., 2007).

Our novel mouse model allows unequivocal analysis of OPN3 protein expression by monitoring mCh fluorescence in an entire mouse. Consequently, our first endeavor with this mouse model was to enable the complete characterization of endogenous OPN3 expression in the adult brain. Animals homozygous for the knock-in mCh-tagged *Opn3* allele were used to systematically characterize OPN3-mCh expression in the adult mouse brain from coronal, sagittal, and horizontal sections. We examined OPN3-mCh-expressing cells for coexpression of neuronal, astrocytic, ChAT, and GABAergic markers to gain further insight into the types of cells expressing OPN3 for future functional studies.

## Materials and Methods

### Generation of the OPN3-mCh knock-in mouse

**Figure 1.**
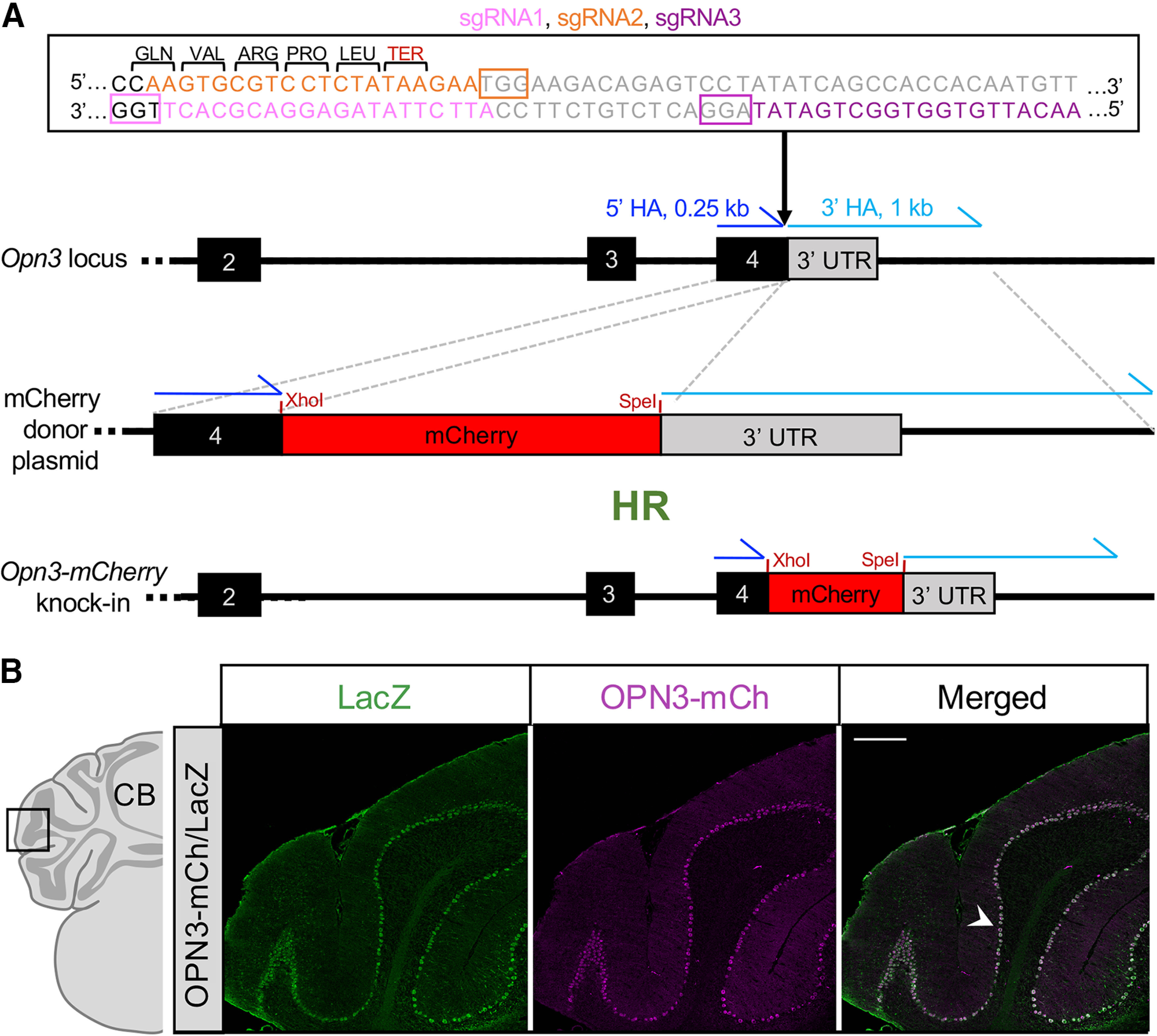
OPN3-mCh knock-in mouse model generation using CRISPR/Cas9 editing. ***A***, Targeting strategy for the OPN3-mCh knock-in allele. mCherry (mCh) was inserted in-frame immediately before the termination codon in exon 4 of mouse *Opn3*. The resulting fusion protein expresses mCh fused at the C terminus of OPN3 via a short linker. Double-stranded DNA sequence highlights sgRNA1 (pink), sgRNA2 (orange), sgRNA3 (purple), *Opn3* exon 4 (black), and *Opn3* 3′ UTR (gray) with colored boxed areas indicating PAM sites. HR: homologous recombination, HA: homology arm, TER: termination codon. Primers for genotyping are shown in Extended Data Figure 1-1. ***B***, Validation of OPN3-mCh brain expression using the OPN3-mCh/LacZ mouse. The OPN3-mCh/LacZ mouse was generated by crossing our OPN3-mCh knock-in mouse with a mouse in which OPN3 was replaced by LacZ (OPN3^LacZ/LacZ^; Buhr et al., 2015). A representative brain section of the cerebellum (CB) immunostained for OPN3-mCh and LacZ shows significant overlap in Purkinje cells (arrowhead). Scale bar: 250 μm.

In conjunction with the Brown University Mouse Transgenic and Gene Targeting Facility, CRISPR/Cas9 gene editing technology was used to insert mCherry (mCh) in-frame at the carboxy (C) terminus of the endogenous *Opn3* locus, before the termination codon. The resultant knock-in mouse model expresses the fusion protein OPN3-mCh under the control of the endogenous *Opn3* promoter. The Transgenic Facility microinjected >200 C57BL/6 embryos with 10 ng/μl each of (1) sgRNA1/sgRNA2 or sgRNA1/sgRNA3 (Table 2: Primers and sgRNA sequences**;** made in-house through *in vitro* transcription with MEGAshortscript T7 Transcription kit, Ambion), which target nucleotides immediately surrounding the *Opn3* termination codon; (2) Cas9 mRNA (Alt-R S.p. Cas9 Nuclease V3, IDT); and (3) a donor plasmid comprised of a pUC57 backbone (Addgene) with a 0.25 kb 5′ homology arm followed by a linker (LEGAGA), mCh, then a 1.0 kb 3′ homology arm (Fig. 1*A*). Embryos were implanted in the oviducts of pseudopregnant Swiss Webster females (Taconic Biosciences). Two G_0_ founders (one from sgRNA1/sgRNA2 and one from sgRNA1/sgRNA3) were confirmed to have mCh inserted in-frame by long-range and short-range PCR. G_0_ founders were crossed with C57BL/6 mice to obtain a stock of G_1_ OPN3-mCh/+ heterozygotes. After backcrossing, heterozygous OPN3-mCh/+ mice were interbred to obtain homozygous OPN3-mCh/OPN3-mCh mice (herein referred to as OPN3-mCh mice) which were confirmed by short-range PCR. Neither the heterozygous nor homozygous OPN3-mCh mice have any observable phenotype. We did not observe any differences in OPN3-mCh localization between the heterozygote and the homozygote, only differences in mCh fluorescence levels, as expected. Thus, we performed all staining experiments (unless otherwise stated) using sections from homozygous mice because the fluorescence intensity was greater. OPN3-mCh male and female mice are fertile, but as with any genetically-modified animal, breeding efficiency in the OPN3-mCh mice is less robust than in wild-type (WT) C57BL/6 mice. The OPN3-mCh female mice tend to have smaller litters (approximately two to four pups), and we have observed them to be more prone to cannibalizing their young.

10.1523/ENEURO.0107-20.2020.f1-1 Extended Data Figure 1-1 OPN3 mouse primers for identification and genotyping. Primers used to verify mCherry insertion and for subsequent genotyping. Primer names and pairs correspond to Materials and Methods, Primers and sgRNA sequences. LR-PCR: long-range PCR, SR-PCR: short-range PCR Download Figure 1-1, TIF file.

### Origin of other mouse lines used

C57BL/6 mice originated from The Jackson Laboratory. The OPN3 knock-out (OPN3^LacZ/LacZ^) transgenic mice (Buhr et al., 2015) were a generous gift from K.-W. Yau at Johns Hopkins University. In brief, this line was generated by Buhr et al. (2015) by crossing OPN3^flox/flox^ mice with *Sox2-Cre* mice. OPN3^flox/flox^ mice contained a targeting construct for inserting a flippase recognition target-flanked LacZ cassette upstream of *Opn3* exon 2. Cre-recombination led to global deletion of endogenous *Opn3* and insertion of LacZ under the *Opn3* promoter and thus creation of the OPN3^LacZ/LacZ^ line.

### Animals

All animal care and procedures in this study were performed in accordance with the Brown University animal care committee’s regulations. Animals were housed socially with *ad libitum* access to chow and water in a light-controlled (12/12 h light/dark cycle) and temperature-controlled (21.5–23.5°C) facility. Both male and female mice were used, as there was no significant difference in OPN3 expression between sexes. Genotyping primers for OPN3-mCh and OPN3^LacZ/LacZ^ are in Table 2: Primers and sgRNA sequences; and Extended Data Figure 1-1.

### Tissue collection

Ten mice (4–12 months old; five OPN3-mCh, three WT, two OPN3-mCh/LacZ) were deeply anesthetized with 100 mg/kg ketamine and 0.25 mg/kg dexmedetomidine via subcutaneous injection and transcardially perfused with 50 ml of ice-cold PBS, followed by 50 ml of freshly prepared, ice-cold 4% paraformaldehyde. Once brains were removed, they were postfixed in 4% paraformaldehyde for <18 h at 4°C. After rinsing thoroughly with cold PBS, brains were cryoprotected in increasing concentrations of sucrose-PBS solutions (10%, 20%, 30%) over 3 d (or until the brain sank to the bottom of the holding container). Brains were flash-frozen for 10–15 s in a slurry of 2-methylbutane and dry ice and stored at −80°C until sectioning on a cryostat (Leica CM3050S) at 25–30 μm. Sections were stored in an antifreeze solution [20% (v/v) glycerol, 20% (v/v) ethylene glycol in 1× PBS] at −20°C until immunostaining.

### Free-floating immunofluorescence

All antibodies and concentrations can be found in Table 1: Antibodies Brain sections were washed with PBS 2 × 5 min then permeabilized with 0.3% PBS-Triton X-100 (PBST) 3 × 10 min. Brains were blocked with a solution of 5% donkey serum and 1% BSA in PBS for ∼2 h at room temperature (RT). Sections were incubated in primary antibody diluted in the above blocking solution for 24–72 h at 4°C with light shaking (50–70 rpm). Sections were then washed in PBS 3 × 10 min before blocking/permeabilizing in a solution of 5% donkey serum in 0.3% PBST for ∼2 h at RT (this step was omitted for MAP2, GAD67, and GFAP immunostainings). Sections were incubated in secondary antibody diluted in 0.3% PBST for ∼2 h at RT with light shaking. After a final wash in PBS 3 × 10 min and a <1-min rinse in deionized water, the sections were placed on SuperFrost Plus slides (Thermo Fisher Scientific) and mounted with VECTASHIELD Antifade Mounting Media with DAPI (Vector Laboratories). For Nissl stains, free-floating sections were rinsed 3 × 5 min in PBS before staining with NeuroTrace 530/615 Red Fluorescent Nissl Stain (Thermo Fisher Scientific) according to manufacturer’s instructions (omitting initial rehydration step). Sections were mounted as noted above.

### Microscopy, image acquisition, and analysis

**Figure 2.**
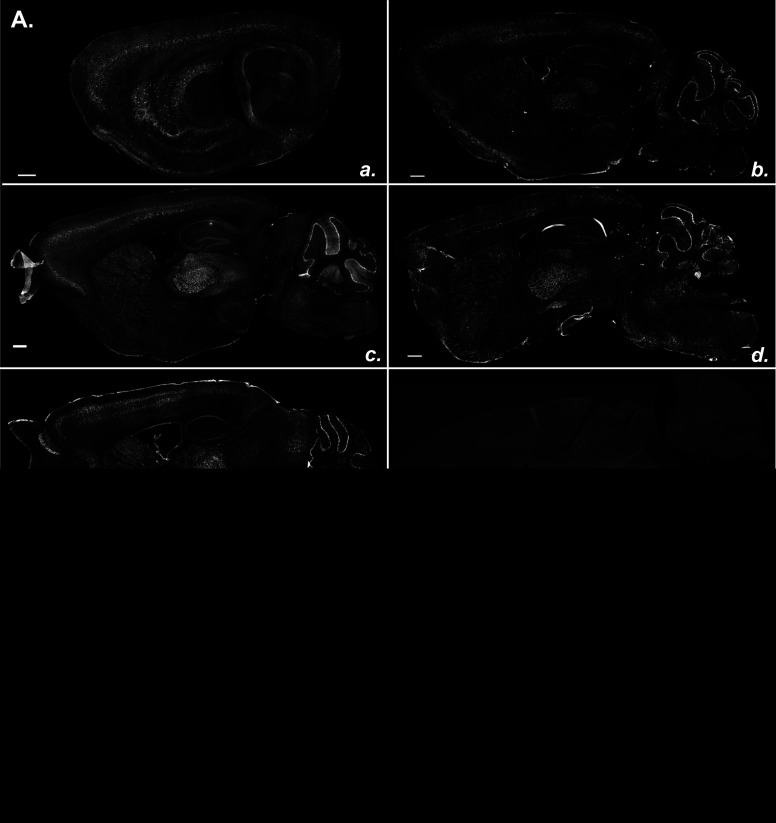
Endogenous OPN3-mCh expression across the adult OPN3-mCh mouse brain. ***Aa–Ae***, Fluorescent images of sagittal brain sections from adult OPN3-mCh mice, immunostained with anti-mCh antibody and arranged in a lateral-to-medial manner. Note high OPN3-mCh expression in the thalamus, cerebellum, HCF, and CTX. ***Af***, Representative fluorescent image of a sagittal brain section from a WT mouse immunostained with anti-mCh antibody, imaged under the same settings as the sections in ***Aa–Ae***; it has negligible fluorescence signal, as expected. Scale bars: 500 μm. ***Ba–Bd***, Fluorescent images of horizontal brain sections from adult OPN3-mCh mice immunostained with anti-mCh antibody and arranged in a dorsal-to-rostral manner. Bright circle in left midbrain of ***Bb*** is an imaging artifact. Scale bars: 500 μm.

**Figure 3.**
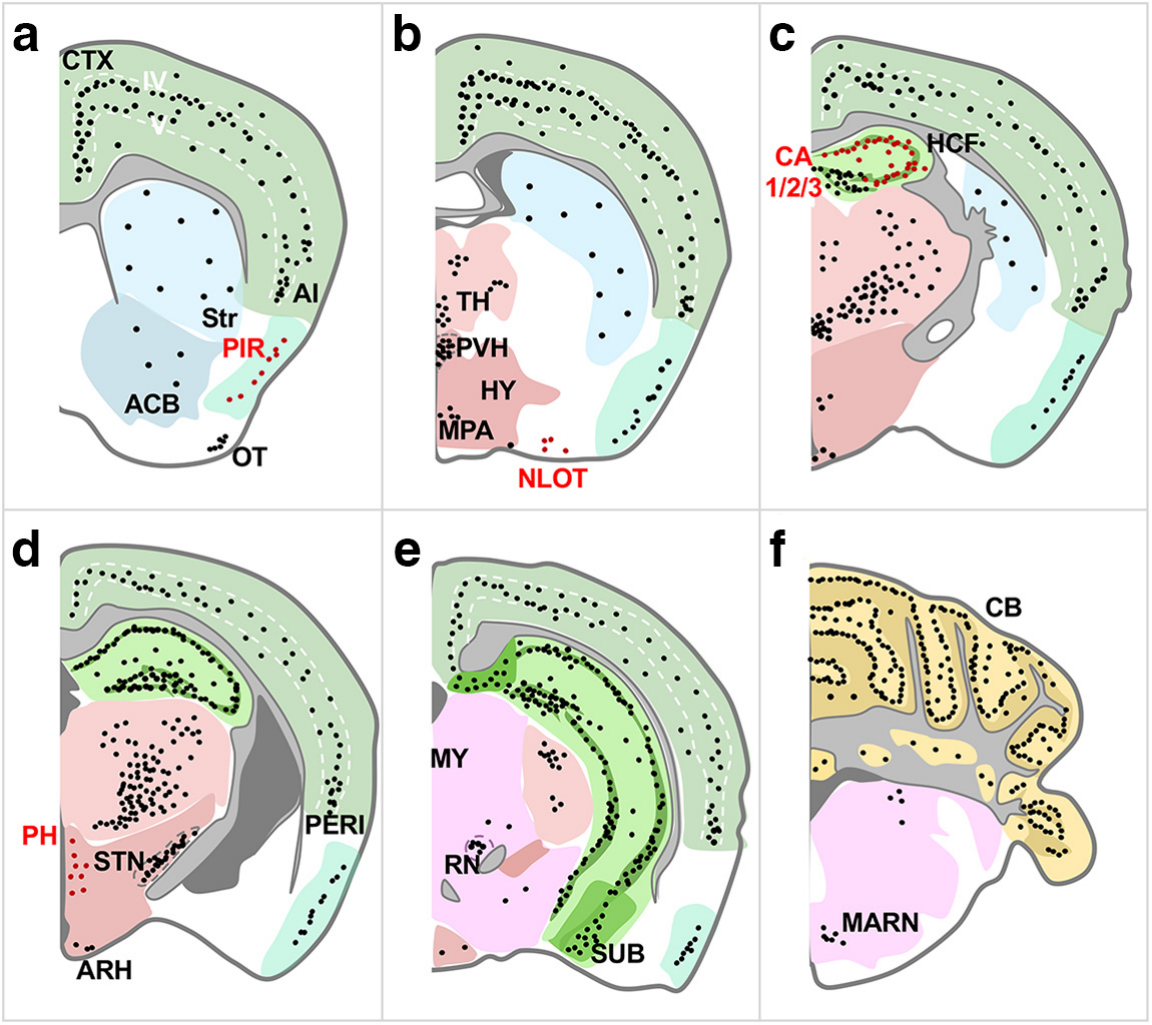
Semi-quantitative analysis of OPN3 distribution and abundance in the adult OPN3-mCh mouse brain. ***a–f***, Line drawings of coronal sections arranged in a rostral-to-caudal manner with each black dot representing ∼10 OPN3-mCh-positive cells, averaged from coronal, sagittal, and horizontal sections from four OPN3-mCh mice immunostained and imaged as in Figure 2. Red labels and dots indicate new regions of OPN3 expression revealed by our OPN3-mCh mouse. Different colors correspond to different brain regions as labeled; dark gray corresponds to ventricles and light gray to fiber tracts. As indicated by the density of black dots, high expression of OPN3-mCh was detected in Layers IV–V of the CTX, HCF, ventral and lateral nuclei of the thalamus (TH), and cerebellum (CB). Representative fluorescent images of coronal sections used for analysis are shown in Extended Data Figure 3-1. ACB: nucleus accumbens, AI: agranular insular area, ARH: arcuate nucleus, HY: hypothalamus, MARN: magnocellular reticular nucleus, MY: medulla, NLOT: nucleus of the lateral olfactory tract, OT: olfactory tubercle, PERI: perirhinal area, PH: posterior hypothalamic nucleus, PIR: piriform area, RN: red nucleus, STN: subthalamic nucleus, Str: striatum, SUB: subiculum.

**Figure 4.**
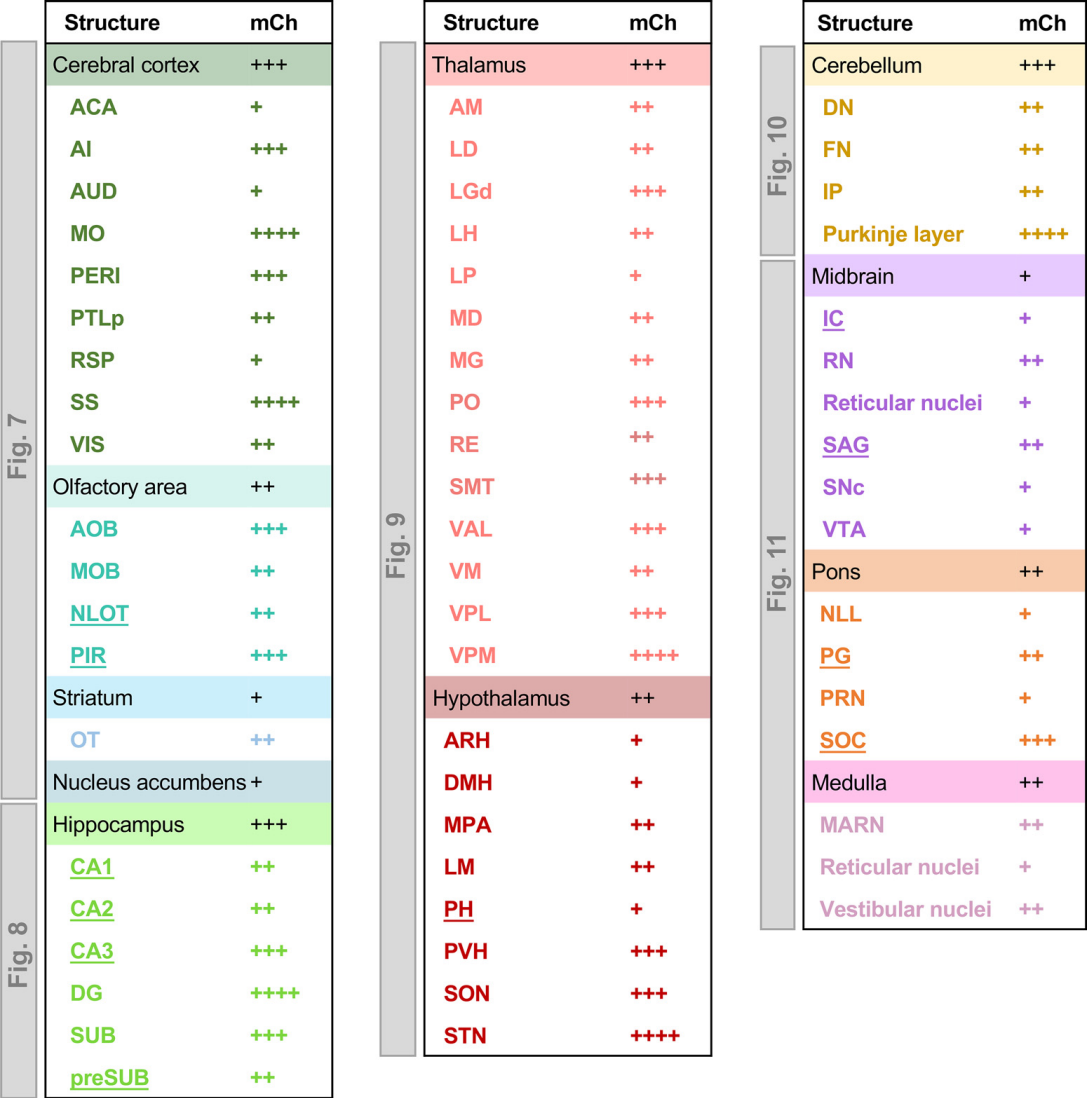
R>elative abundance of OPN3-mCh immunoreactivity in different regions of the OPN3-mCh mouse brain. Estimated abundance of OPN3-mCh-expressing cells in each brain subregion was based on the fraction of mCh immunoreactive cells over total cells visualized by DAPI in coronal, sagittal, and horizontal sections across the adult OPN3-mCh mouse brain. ++++: highest density, +++: high density, ++: moderate density, +: low density. Newly identified brain areas expressing OPN3-mCh are underlined. Similarities and differences of OPN3 expression between our identified brain regions and previous reports are in Extended Data Figure 4-1. ACA: anterior cingulate area, AI: agranular insular area, AM: anteromedial nucleus, ARH: arcuate nucleus, AUD: auditory area, DMH: dorsomedial nucleus, DN: dentate nucleus, FN: fastigial nucleus, IC: inferior colliculus, IP: interposed nucleus, LGd: dorsal lateral geniculate nucleus, LH: lateral habenula, LM: lateral mammillary nucleus, MARN: magnocellular reticular nucleus, MD: mediodorsal nucleus, MG: medial geniculate complex, MO: somatomotor area, MOB: main olfactory bulb, NLL: nucleus of the lateral lemniscus, NLOT: nucleus of the lateral olfactory tract, OT: olfactory tubercle, PG: pontine grey, PERI: perirhinal area, PH: posterior hypothalamic nucleus, PIR: piriform area, preSUB: presubiculum, PRN: pontine reticular nucleus, PTLp: posterior parietal association area, RE: nucleus of reuniens, RN: red nucleus, RSP: retrospenial area, SAG: nucleus sagulum, SNc: substantia nigra compacta, SON: supraoptic nucleus, SOC: superior olivary complex, SS: somatosensory area, STN: subthalamic nucleus, SUB: subiculum, VIS: visual area, VTA: ventral tegmental area.

Fluorescence staining was viewed using either an Olympus FV3000 Confocal Laser Scanning Microscope, Zeiss LSM 800 Confocal Laser Scanning Microscope or Nikon Ti2-E Fluorescence Microscope. The Olympus FV3000 has an inverted configuration and is equipped with a resonant scanner and four detectors, two standard Metal Alkalide and two high-sensitivity GaAsP. The Zeiss LSM 800 is based on an Axio Imager Z2 microscope and is equipped with a three high-sensitivity GaAsP detectors. Widefield images in Figure 2 were acquired on the Nikon Ti2-E microscope using a 10× objective and stitched using Nikon-based NIS-Elements software. Zoomed regions were acquired on the Olympus FV3000 or Zeiss LSM 800 microscopes using 10–30× objectives and stitched using cellSens (Olympus) or Zen Blue (Zeiss) software. The images from OPN3-mCh mice and WT control mice were collected using identical acquisition settings. FIJI/ImageJ (NIH) was used to adjust image brightness/contrast, using the same parameters for images obtained from OPN3-mCh and WT control mice. Scale bars were added to final images using FIJI/ImageJ. The density of OPN3-mCh-expressing cells by area in Figure 3 was determined based on serial coronal, sagittal, and horizontal sections from four OPN3-mCh mice. The number of positive OPN3-mCh cells was determined using DAPI counterstain via visual analysis. Brain regions were named using conventions from Franklin and Paxinos (Paxinos and Franklin, 2013). Anatomy outlines, and color schemes in figures are based on the Allen Mouse Brain Atlas (Lein et al., 2007). Estimates of the abundance of OPN3-mCh-expressing cells in Figure 4 were determined from both widefield and confocal images in coronal, horizontal, and sagittal planes. Image contrast was adjusted and a merged image of the DAPI channel on the mCh channel was used. Abundance was determined through visual analysis of mCh positive cells over total cell number per brain region per section. The relative abundance of OPN3-mCh cells was noted as very high: ++++; high: +++; moderate: ++; or low: +, corresponding approximately to the following percentages of OPN3-mCh+ cells/total cells: very high: >70%; high: 40–70%; moderate: 10–40%; low: 5–10%.

**Table 1:** Antibodies

Antibody	Company	Concentration
Anti-mCherry	KeraFAST (EMU105)	1:500
Anti-β-galactosidase (LacZ)	GeneTex (GTX77365)	1:500
Anti-MAP2	Santa Cruz Biotechnology (sc-74421)	1:200
Anti-GFAP	Santa Cruz Biotechnology (sc-33673)	1:200
Anti-GAD67	Millipore Sigma (MAB5406)	1:500
Anti-ChAT	Millipore Sigma (AB144P)	1:500
Goat anti-mouse IgG2a-568	Life Technologies (A21134)	1:500
Donkey anti-mouse-647	Invitrogen (A31571)	1:500
Donkey anti-rabbit-488	Invitrogen (A32814)	1:500
Donkey anti-rabbit-594	Invitrogen (A32754)	1:500
Donkey anti-chicken-647	Jackson ImmunoResearch (703-605-155)	1:500

**Table 2:** Primers and sgRNA sequences

Sequence	Use
ATTCTTATAGAGGACGCACT	sgRNA1
AAGTGCGTCCTCTATAAGAA	sgRNA2
AACATTGTGGTGGCTGATAT	sgRNA3
CTCCGTTGTTTCTCTCTGCAG	Primer 360 Full length PCR-Forward
ACGCCAGATGCTCATCTTG	Primer 362 OPN3 WT-Reverse
CTCAGTGACTTCCAACTCAAGG	Primer 367 Full length PCR-Reverse
CTCTACTCGAGGGAGCAGGAG	Primer 369 3’ Long Range PCR-Forward
AAGCGCATGAACTCCTTGATG	Primer 371 OPN3-mCh-Reverse
CTTGTACAGCTCGTCCATGC	Primer 372 5’ Long Range PCR-Reverse
TTATGGCCCACACCAGTGGC	Primer OPN3 KO-Forward
TGTACCGTGGACTGGAGATCCAAG	Primer OPN3 WT-Forward
GTTCCCACACACGACCTGCTC	Primer OPN3-Reverse

10.1523/ENEURO.0107-20.2020.f3-1 Extended Data Figure 3-1 Representative coronal sections from the OPN3-mCh mouse used for the semi-quantitative analysis in Figure 3. Fluorescent images of coronal sections from homozygous OPN3-mCh mice, roughly corresponding to the planes represented in Figure 3. OPN3-mCh is in magenta and DAPI counterstain is in blue. All scale bars: 500 μm. Download Figure 3-1, TIF file.

10.1523/ENEURO.0107-20.2020.f4-1 Extended Data Figure 4-1 Comparison of previously reported OPN3 expression to our OPN3-mCh protein expression. Previously published or publicly accessible OPN3 mRNA or protein expression in the mouse brain as compared to our OPN3-mCh expression data by structure. In red are newly identified areas of OPN3 expression revealed by the OPN3-mCh mouse. In blue are areas of OPN3 expression found previously but not detected by the current OPN3-mCh analysis. Allen Brain Atlas ISH Data and GENSAT subregions were interpreted from publicly available brain sections from C57BL/6J (P56) and Tg(Opn3-EGFP)JY3Gsat/Mmucd (P7, adult) mice, respectively. *, OPN3 mRNA; +, endogenous OPN3 protein; ++, reporter for OPN3. Download Figure 4-1, TIF file.

## Results

### Generation of the OPN3-mCh mouse

The OPN3-mCh knock-in mouse was generated using CRISPR/Cas9 technology for efficient and precise insertion of the OPN3-mCh fusion protein under the endogenous *Opn3* promoter. A combination of two sgRNAs targeting the junction between the last exon of mouse *Opn3* (exon 4) and the 3′ untranslated region (UTR) were injected along with Cas9 mRNA and a donor plasmid containing mCh into C57BL/6 embryos (Fig. 1*A*). A more extensive description of procedures can be found in Materials and Methods. The fluorescent tag mCh was chosen over other red fluorophores (such as tdTomato) based on availability of reliable antibodies against mCh, in the event that fluorescent expression on its own was too dim to detect. We used the same OPN3 fusion protein construction (mCh attached to the C terminus of OPN3 via a flexible linker) that was previously demonstrated not to interfere with localization or function of the OPN3 protein in melanocytes (Ozdeslik et al., 2019). After confirming successful knock-in of mCh at the direct C terminus of *Opn3*, heterozygous mice were backcrossed and bred to create mice homozygous for OPN3-mCh. Homozygous OPN3-mCh mice were used for experiments unless stated.

The endogenous mCh fluorescence in brain sections from OPN3-mCh mice was too weak to detect. We therefore used a validated anti-mCh antibody to amplify the endogenous mCh signal as a proxy for OPN3-mCh expression. The same anti-mCh antibody was used for immunostaining skin sections from OPN3-mCh mice and showed a detectable and reliable signal in cells known to express OPN3, but not in control mice without OPN3- mCh (Olinski et al., 2020). We next sought to confirm the fidelity of OPN3-mCh expression to endogenous OPN3 expression in the brain. In previous publications we used an OPN3 antibody to detect endogenous OPN3 (Ozdeslik et al., 2019), but the discontinuation of this antibody and lack of a suitable replacement led us to pursue an alternative approach to confirm that OPN3-mCh in the knock-in mouse can successfully be detected in areas where endogenous OPN3 is known to be expressed. We crossed the previously generated OPN3 β galactosidase (LacZ)-reporter mouse (OPN3^LacZ/LacZ^; Buhr et al., 2015) with our OPN3-mCh knock-in mouse to create OPN3-mCh/LacZ mice. These mice, under the *Opn3* promoter, expressed one LacZ allele and one OPN3-mCh allele. Figure 1*B* is a representative image of the cerebellum of the OPN3-mCh/LacZ mouse coimmunostained for LacZ and mCh showing LacZ and mCh signal overlap and specificity to Purkinje cells of the cerebellum. This indicates that the OPN3-mCh expression in the OPN3-mCh mouse line is localized consistently with the *Opn3* promoter-controlled LacZ reporter, as expected. Because this was a heterozygous mouse for the OPN3-mCh allele, the fluorescence expression was lower than for the homozygous mice and thus was best in brain areas with high OPN3 expression, namely, the cerebellum. We also used the cerebellum to test specificity because this region is undisputed as an area with high OPN3 expression in the brain (Blackshaw and Snyder, 1999; Lein et al., 2007; Nissilä et al., 2012). For a more complete characterization of OPN3 expression in the brain, we used homozygous OPN3-mCh mice to identify and estimate the expression level of OPN3 in different brain areas. To ensure the specificity of the mCh antibody, we immunostained brain sections from OPN3-mCh and WT mice. We saw specific and consistent expression of mCh across serial sections from OPN3-mCh mice and no mCh immunoreactivity in WT sections (Fig. 2). OPN3-mCh sections in the following figures were stained with anti-mCh antibody to amplify OPN3-mCh signal and matching WT brain slices were used as controls for all experiments.

### Endogenous OPN3-mCh is present in several brain regions in neuronal and non-neuronal cell types

Sagittal and horizontal OPN3-mCh brain sections revealed unique patterns of expression (Fig. 2). We obtained sagittal (Fig. 2*A*), horizontal (Fig. 2*B*), and coronal (Fig. 3; Extended Data Fig. 3-1) sections from OPN3-mCh mouse brains to perform semi-quantitative analysis of OPN3-mCh expression in individual regions. Figure 3 shows coronal depictions of OPN3-mCh expression profiles in which the density of the black dots (each representing ∼10 OPN3-mCh cells) shows distinct patterns of known expression across regions. Areas of new OPN3 expression identified using our OPN3-mCh mouse are noted in red (Fig. 3). Representative fluorescent images can be found in Extended Data Figure 3-1. OPN3-mCh exhibited a double-banded pattern in the CTX, most prominently within Layers IV and V; sparse, but even distribution within the striatum; ventral-specific and lateral-specific expression in the thalamus; and robust expression in the Purkinje cell layer of the cerebellum. These observations are summarized according to the mCh immunostaining intensity of each region in Figure 4.

**Figure 5.**
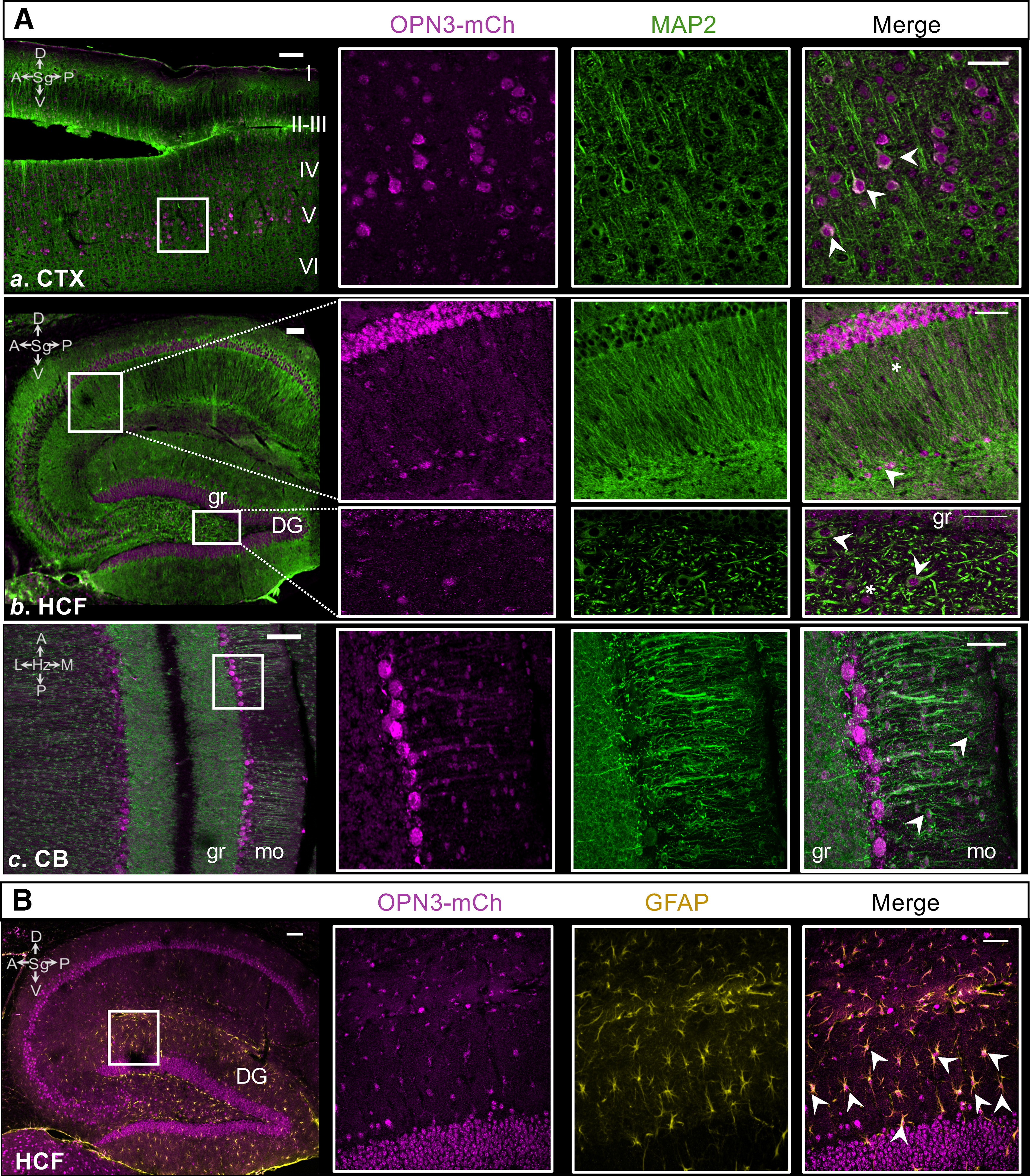
Relative distribution of OPN3 and neuronal (MAP2) or astrocytic (GFAP) markers in the adult OPN3-mCh mouse brain. ***A***, Colocalization of OPN3-mCh (magenta) and MAP2 (green). ***Aa***, Large, pyramidal neurons in Layer V of the CTX express OPN3-mCh and the neuronal marker MAP2. ***Ab***, In the HCF, OPN3-mCh-expressing cells overlap with MAP2 in the granule layer (gr) and in some larger cells at the junction of CA1 and DG (arrowhead). Some smaller, multidendritic OPN3-mCh cells appear not to contain MAP2 (asterisk). ***Ac***, Purkinje cells in the cerebellum (CB) coexpress both MAP2 and OPN3-mCh; OPN3-mCh is present in some granule neurons (gr) and many molecular layer (mo) neurons (arrowheads). ***B***, Colocalization of OPN3-mCh (magenta) and GFAP (yellow) in the HCF. Many OPN3-mCh-expressing cells in the HCF with star-like morphology are also immunostained for GFAP, indicating that especially in the DG, astrocytes express OPN3-mCh (arrowheads). Initial zoomed out images for each region have scale bars: 100 μm. All magnified boxed regions are maximal projections from z-stacks with scale bars: 50 μm.

Our first objective was to broadly determine in which cell types OPN3-mCh is expressed. From other studies, it was known that OPN3 is expressed in at least some neurons, like the Purkinje cells of the cerebellum. It was unknown, however, whether OPN3 was also present in non-neuronal cells within the brain. As we conducted our analysis of the OPN3-mCh brain, it soon became evident that OPN3 is expressed in non-neuronal cell types, notably in the hippocampal formation (HCF). To determine neuronal expression of OPN3-mCh, we coimmunostained sections with mCh and the neuronal marker microtubule-associated protein 2 (MAP2; Cassimeris and Spittle, 2001). Figure 5 shows representative examples of three brain regions with neuronal overlap: the CTX, cerebellum, and HCF. Within the CTX, OPN3-mCh was present in large, pyramidal neurons in the somatomotor area, Layer V (Fig. 5*Aa*). In the cerebellum, there was strong neuronal expression of OPN3-mCh in the Purkinje cell layer, molecular layer, and to a lesser extent, the granule layer (Fig. 5*Ac*). Expression in the HCF was less homogenous with regard to cell type. OPN3-mCh staining was apparent in the pyramidal cell layers in Ammon’s horn (CA) CA1/2/3 and the granule layer and hilus of the dentate gyrus (DG; Fig. 5*Ab*) as well as small cells with multiple processes that were not clearly labeled by MAP2 (Fig. 5*Ab*). These are likely astrocytes, as they express glial fibrillary acidic protein (GFAP; Messing and Brenner, 2003; Fig. 5*B*); OPN3-mCh colocalized with GFAP in most of the GFAP+ astrocytes. This is the first instance of OPN3 identification in a non- neuronal cell type.

**Figure 6.**
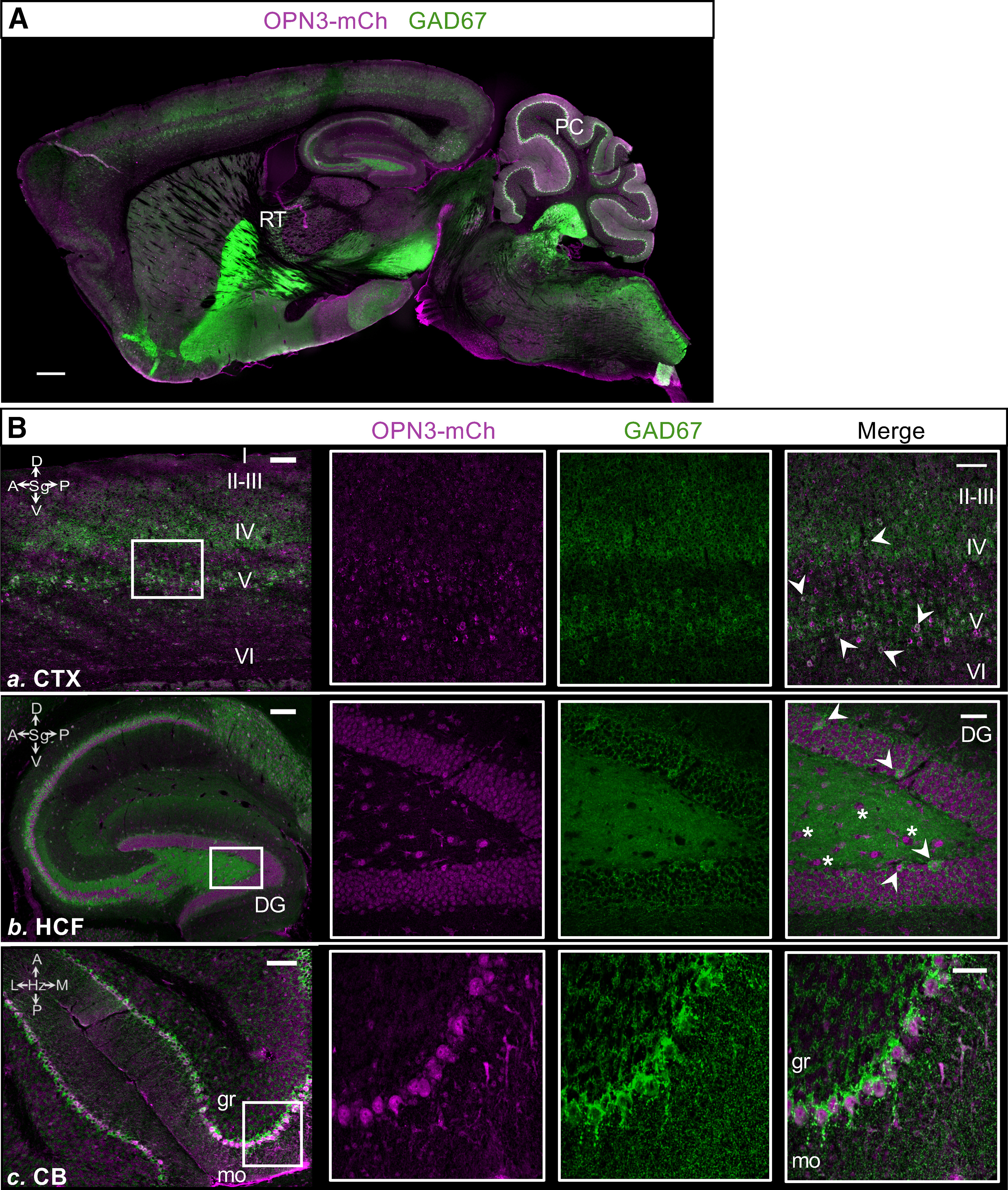
Relative distribution of OPN3 and GAD67 in the adult OPN3-mCh mouse brain. ***A***, Distribution of OPN3-mCh (magenta) and GAD67 (green) in a representative sagittal section of the adult OPN3-mCh brain. Scale bar: 500 μm. PC: Purkinje cell layer of the cerebellum, RT: reticular nucleus of the thalamus. ***B***, Colocalization of OPN3-mCh and GAD67. ***Ba***, Many OPN3-mCh cells are GAD67+ in Layers IV–V and at the V–VI border (arrowheads) of the CTX. ***Bb***, In the HCF, nearly all GAD67+ cells also express OPN3-mCh (arrowheads). But, especially in the DG, not all OPN3 mCh cells are GAD67+ (asterisks). ***Bc***, Purkinje cells are GAD67+ and express OPN3-mCh. OPN3-mCh is present in some, but not all GAD67+ cells within the granule (gr) and molecular (mo) layers of the cerebellum (CB). Initial zoomed out images for each region have scale bars: 100 μm (***a***, CTX), 200 μm (***b***, HCF), 100 μm (***c***, CB). All magnified boxed regions are maximal projections from z-stacks with scale bars: 50 μm.

We also noticed that many of the regions in which OPN3 is expressed (Figs. 2-4) are known to contain populations of GABAergic cells (Tamamaki et al., 2003): the CTX, cerebellum, and striatum, among other regions. To further dissect whether OPN3-mCh is found preferentially in GABAergic cells, we stained OPN3-mCh sections for glutamic acid decarboxylase 67 (GAD67), one of two enzymes that catalyze the conversion of glutamate to GABA (Pinal and Tobin, 1998; Fig. 6). GAD67 was concentrated primarily in the soma as previously described (Asada et al., 1997; Kanaani et al., 2010), similar to OPN3-mCh expression. A widefield view of the coimmunostaining indicated that some OPN3-mCh+ cells were also GAD67+, such as cells in the Purkinje layer of the cerebellum. However, not all GAD67+ cells were OPN3-mCh+, as evidenced by the GABAergic reticular nucleus of the thalamus, which lacks OPN3-mCh expression (Fig. 6*A*). At greater magnification in the CTX, Layers IV and V showed strong signal overlap between GAD67 and OPN3-mCh. OPN3-mCh was also present to a lesser extent in Layers II–III and VI, which have less GAD67 expression (Fig. 6*Ba*). In the HCF, GAD67 staining revealed that OPN3-mCh was present in GAD67+ cells around the pyramidal and granule layers. Within the DG, OPN3-mCh was expressed in GAD67+ cells, but also in cells devoid of GAD67, putatively glutamatergic mossy cells in the hilus (Fig. 6*Bb*). As expected from previous studies, OPN3-mCh was also present in GABAergic Purkinje cells and in many GABAergic cells of the molecular layer of the cerebellum (Fig. 6*Bc*). Because it was difficult to detect OPN3-mCh expression in terminals and GAD65, the other enzyme that catalyzes the conversion of glutamate to GABA is generally concentrated in axon terminals (Kaufman et al., 1991), we could not evaluate the overlap between GAD65 and OPN3-mCh. Future work investigating the overlap of all GABAergic neurons, identified by GAD65 and GAD67 immunoreactivity, with OPN3 expression may be beneficial to fully understand the functions of OPN3.

### Endogenous OPN3-mCh expression in individual brain regions

To further examine expression patterns of OPN3-mCh and the morphology of these cells, we next looked at OPN3-mCh expression by region.

#### Olfactory area, CTX, and striatum

**Figure 7.**
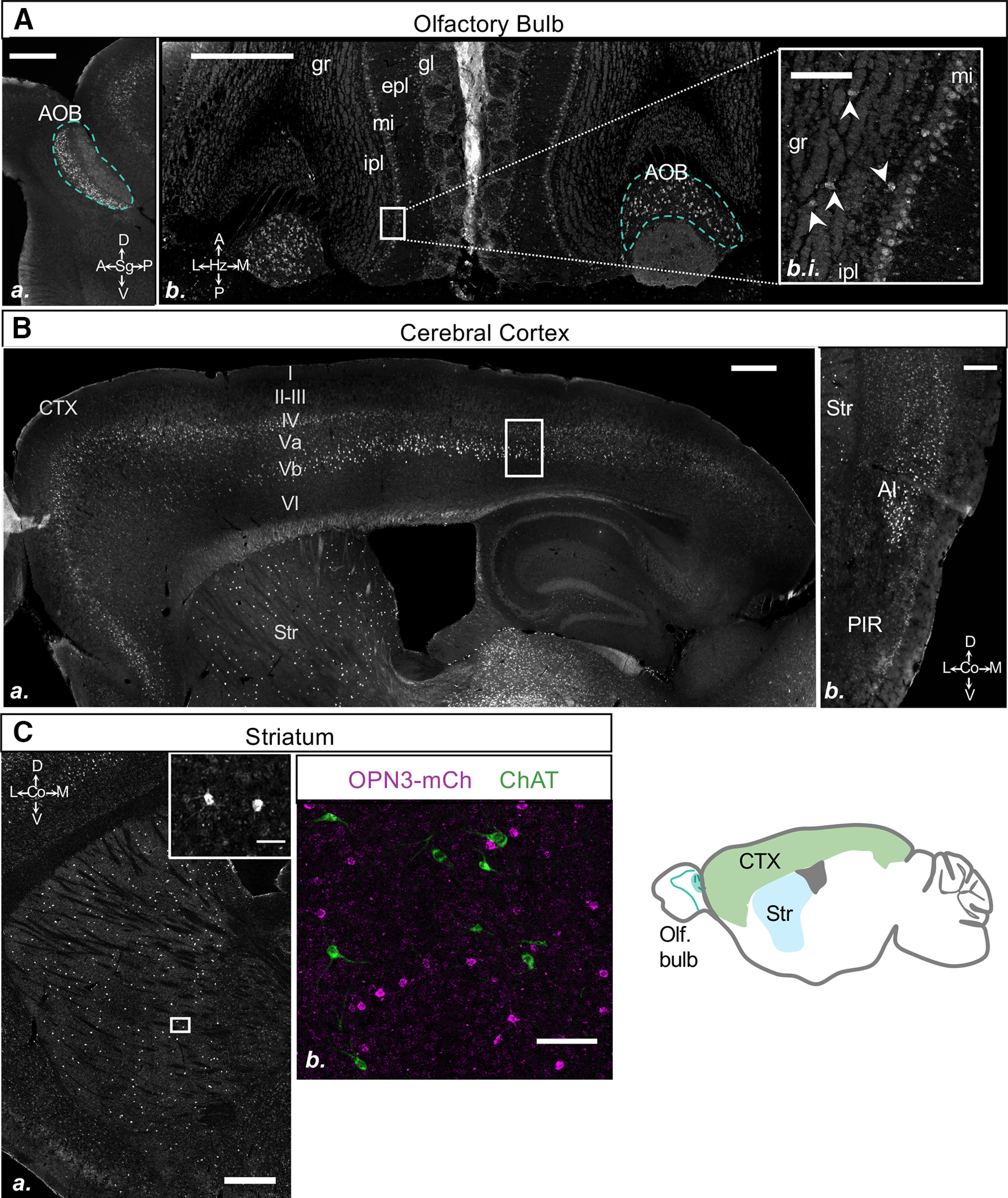
OPN3 expression in the olfactory bulb, CTX, and striatum of the adult OPN3-mCh mouse brain. ***Aa***, There is high OPN3-mCh expression in the AOB granular layer (outlined in turquoise). ***Ab***, OPN3-mCh expression in the AOB, and mitral cell (mi), glomerular (gl), granule (gr), and external plexiform (epl) layers; almost no expression in inner plexiform layer (ipl). ***Abi***, Magnified image of the boxed region of the mitral cell layer in ***Ab***. Arrowheads denote OPN3-mCh expression in certain cells of the granule (gr) as well as many cells of the mitral (mi) layers. Scale bars: 500 μm (***Aa***, ***Ab***) and 100 μm (***Ac***). ***Ba***, Expression of OPN3-mCh in two distinct layers of the CTX. Layers III–VI contain OPN3-mCh with the strongest expression in Layers IV and Vb. ***Bb***, Strong OPN3-mCh expression is present in the agranular insular area (AI) and piriform area (PIR), Layer II. Scale bar: 250 μm. Nissl counterstain is shown in Extended Data Figure 7-1. ***Ca***, OPN3-mCh expression in a subset of small, dendritic cells in the striatum (Str). Scale bar: 500 μm. Inset, OPN3-mCh cells have several proximal dendrites. Scale bar: 60 μm. ***Cb***, No overlap between ChAT positive cells (green) and OPN3-mCh-expressing cells (magenta) in the Str. Scale bar: 100 μm.

There was robust and dense OPN3-mCh expression in several regions of the olfactory bulb. We observed that the accessory olfactory bulb (AOB) granular layer expressed high levels of OPN3-mCh (Fig. 7*A*). Within the main olfactory bulb, OPN3-mCh was expressed predominantly in the mitral cell layer along with a dispersed expression pattern in the external plexiform layer and around the glomeruli (Fig. 7*Ab*). It was evident under higher magnification that there was a subset of OPN3-mCh+ cells within the granule layer (Fig. 7*Abi*). In addition to the olfactory bulb, OPN3-mCh was also expressed in piriform area, Layer II (Figs. 2-4, 7*Bb*).

Previous reports have indicated that OPN3 mRNA (Blackshaw and Snyder, 1999) and protein (Nissilä et al., 2012) are expressed exclusively in Layers IV–VI of the CTX. We saw a similar pattern of OPN3-mCh expression with Layers IV–V having the most prominent expression, although we did observe sparse and weak expression of OPN3-mCh in Layers I–III as well as VI (Fig. 7*Ba*). Expression was highest in the frontal and cingulate cortices when viewed horizontally (Fig. 2*B*). It was clear from a sagittal view that OPN3-mCh exhibited a double-banded pattern with robust expression in Layer IV and in large pyramidal cells of Layer Vb as sections progressed from lateral to more medial (Fig. 7*B*; see also Fig. 2*A*). OPN3-mCh expression extended from agranular insular, orbital, somatomotor to somatosensory areas where double banding became pronounced through posterior parietal associated areas, with expression decreasing caudally through the visual areas and retrosplenial area. The gradient of decreasing expression along the rostrocaudal axis was also observed in coronal sections, where it became evident that the highest OPN3-mCh expression was in the motor and insular areas with diminishing expression as the brain progressed caudally into the auditory and temporal areas. Interestingly, despite decreasing OPN3-mCh expression in the caudal CTX ∼−2 mm posterior to bregma, there was continued, robust, OPN3-mCh expression in Layer V of the agranular insular and perirhinal areas (Figs. 3-4, 7*Bb*).

When it was first discovered, OPN3 mRNA was detected in small cells within the striatum of the mouse (Blackshaw and Snyder, 1999). Since then, a recent publication investigated OPN3 expression in the macaque striatum (El Massri et al., 2018). This report identified two distinct populations of OPN3-expressing interneurons in the striatum: giant cholinergic interneurons positive for ChAT and smaller, ChAT-negative cells. A larger population of ChAT+ interneurons contained OPN3 than the ChAT-negative interneurons. To evaluate whether OPN3 expression in the mouse was similar to what has been observed in the monkey, we examined the striatum of the OPN3-mCh mouse more closely. We observed strong OPN3-mCh expression in small cells, evenly distributed throughout the striatum (Fig. 7*Ca*). mCh immunoreactivity in these cells was not limited to the soma but was also present in many proximal processes. Unlike in the monkey striatum, however, we did not observe OPN3 colocalization with ChAT (Fig. 7*Cb*). OPN3-mCh-expressing cells were smaller in diameter than ChAT-expressing cells, and we saw no indication that OPN3-mCh is expressed in giant cholinergic interneurons in the mouse striatum. This discrepancy is likely because of species differences: monkeys and other more evolved organisms have a much larger striatum than rodents (Petryszyn et al., 2018), and the mouse has a smaller quantity of ChAT+ interneurons. Over evolutionary time, OPN3 may have developed specific functions in ChAT+ interneurons of higher organisms.

10.1523/ENEURO.0107-20.2020.f7-1 Extended Data Figure 7-1 Fluorescent Nissl staining for anatomical reference. Fluorescent Nissl stain of sections immediately preceding or anteceding sections shown in Figures 7, 9, 11. All scale bars: 500 μm. Download Figure 7-1, TIF file.

#### HCF

**Figure 8.**
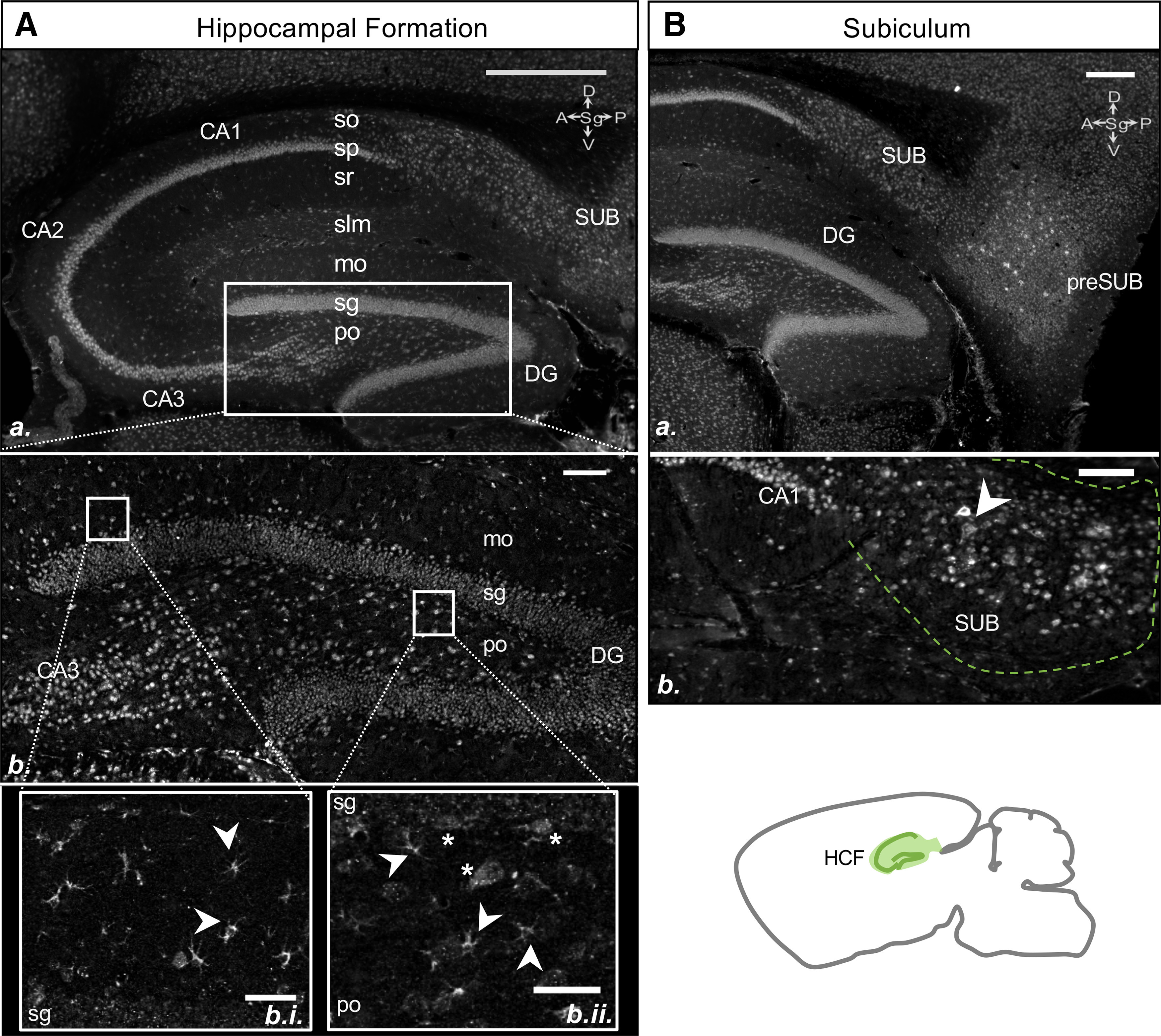
OPN3 expression in the HCF of the adult OPN3-mCh mouse brain. ***Aa***, OPN3*-*mCh in the HCF. Scale bar: 500 μm. ***Ab***, OPN3-mCh in a variety of cell types in all layers of the DG. Scale bar: 100 μm. ***Abi***, Magnified image of DG-mo to DG-sg regions with highly dendritic OPN3-mCh+ cells (with astrocyte-like morphology) indicated by arrowheads. Scale bar: 50 μm. ***Abii***, Magnified image of DG-sg to DG-po regions with highly dendritic astrocytes indicated by arrowheads and larger, triangular neurons indicated by asterisks. Scale bar: 50 μm. ***Ba***, Expression of OPN3-mCh in the subiculum (SUB) and presubiculum (preSUB). Scale bar: 250 μm. ***Bb***, OPN3*-*mCh expression at the plasma membrane and in some proximal dendrites of large cells in the SUB (arrowhead). Scale bar: 100 μm. Layers: mo: molecular, sg: granule, po: polymorph, slm: stratum lacunosum-moleculare, so: stratum oriens, sp: pyramidal cell, sr: stratum radiatum.

Of the previous reports exploring OPN3 in the mouse brain, none have closely examined OPN3 expression in the HCF. Using the OPN3-mCh mouse, we consistently found high expression of OPN3-mCh in the HCF; OPN3-mCh was present in the presubiculum, subiculum, CA1/2/3, and DG (Fig. 8).

Within CA1/2/3, OPN3-mCh was apparent in or around the pyramidal cell layers. Within the stratum oriens, there were large, intensely labeled cells with little dendritic staining along with smaller, extensively branched cells with mCh immunoreactivity in both the soma and dendrites. These two populations were also observed in the stratum radiatum and stratum lacunosum-moleculare, with a higher density of OPN3-mCh in smaller, branched cells within the stratum lacunosum-moleculare than the other stratum layers (Fig. 8*Aa*). Within the DG, OPN3-mCh was observed among all layers: molecular, granule, and polymorph. OPN3-mCh expression in the molecular layer consisted of small, star-like cells throughout (GFAP+ astrocytes; see Fig. 5*B*) and expression in larger, moderately branched cells immediately proximal to the granule layer (Fig. 8*Abi*). The polymorph layer had substantial expression of OPN3-mCh; the morphologies of the OPN3-mCh+ cells indicated that there were different populations expressing OPN3-mCh. Larger, triangular shaped cells at the boundary of the granule and polymorph layers are likely the cell bodies of dentate pyramidal basket cells, which coimmunostain for GAD67 (Figs. 8*Ab*, *Abii*; see also Fig. 6*Bb*). There were also smaller cells with varying degrees of dendritic arborization. Based on coimmuno-staining with GAD67, some appeared to be inhibitory while others that were not stained for GAD67 may instead be mossy cells (Fig. 8*Abii*; see also Figs. 5*B*, 6*Bb*). Within the subiculum and presubiculum, OPN3-mCh expression was robust in a subset of cells (Fig. 8*B*).

#### Thalamus and hypothalamus

**Figure 9.**
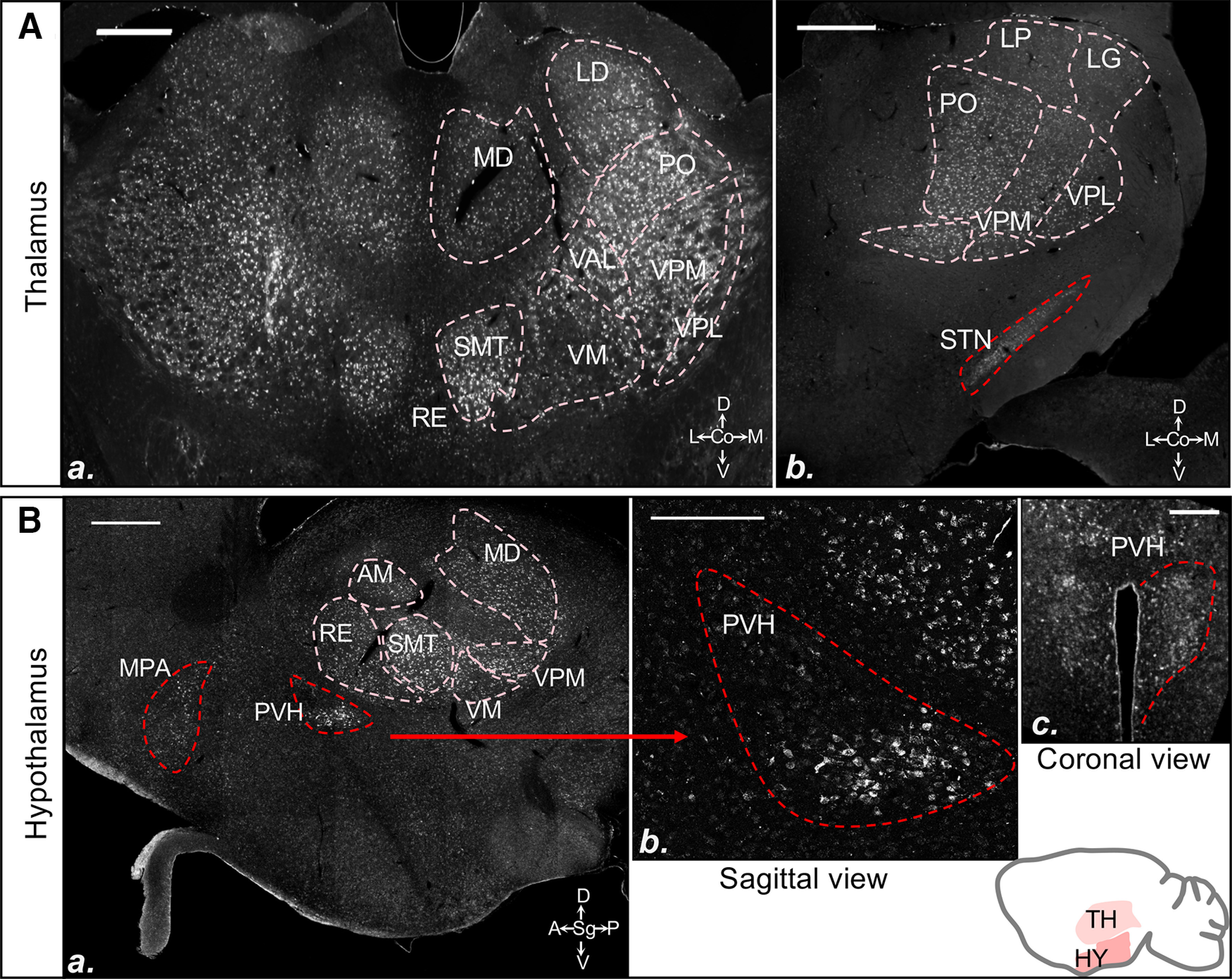
OPN3 expression in the thalamus and hypothalamus of the adult OPN3-mCh mouse brain. ***Aa***, High OPN3-mCh expression in the thalamus (TH), especially in ventromedial and lateral nuclei with moderate expression in the nucleus of reuniens (RE) and mediodorsal nucleus (MD) and low expression in the anteromedial nucleus (AM). ***Ab***, High expression of OPN3-mCh is also detected in the subthalamic nucleus (STN). Scale bars: 500 μm. ***Ba***, Sagittal view of the thalamus and hypothalamus (HY) shows high OPN3-mCh expression in the PVH and moderate expression in the MPA of the hypothalamus. Scale bar: 500 μm. ***Bb***, High density of OPN3-mCh-expressing cells in the PVH. ***Bc***, Coronal view of OPN3-mCh expression in both hemispheres of the PVH. Scale bars: 200 μm (***Bb***, ***Bc***). Pink outline: thalamic nuclei; red outline: hypothalamic nuclei. Nissl counterstains can be found in Extended Data Figure 7-1.

OPN3 mRNA was shown to be highly expressed in the mouse thalamus with a particularly high density in lateral regions (Blackshaw and Snyder, 1999). We saw a consistent pattern, with the thalamus being one of the most OPN3-enriched regions of the brain (Fig. 2). Upon closer inspection, we found OPN3-mCh primarily expressed in ventral, lateral, mediolateral and posterior nuclei with little to no expression in more central regions. Figure 9*A* shows the striking pattern of ventrolateral OPN3-mCh expression. OPN3-mCh was present in the lateral dorsal (LD), lateral geniculate (LG), lateral posterior (LP), posterior (PO), submedial (SMT), ventral posterolateral (VPL), ventral medial (VM), ventral lateral (VAL) and ventral posteromedial (VPM) nuclei. Highest OPN3-mCh expression was observed in the SMT, VAL, VPL, and VPM (Fig. 4).

In the hypothalamus, OPN3 mRNA was initially identified in the PVH and medial preoptic area (MPA) by RNA-seq (Dutra et al., 2020), Western blotting, and immunostaining (Nissilä et al., 2012). We confirmed that OPN3-mCh was expressed in the MPA and highly expressed in the PVH (Fig. 9*B*). RNA-seq has suggested that OPN3 is present in the supraoptic nucleus (Dutra et al., 2020), a region where we also found high OPN3-mCh expression. Additionally, we saw high OPN3-mCh expression in the subthalamic nucleus (Fig. 9*Ab*; see also Fig. 2*A*). Lower expression was identified in the arcuate nucleus, dorsomedial nucleus, and lateral mammillary nucleus (Fig. 4).

#### Cerebellum

**Figure 10.**
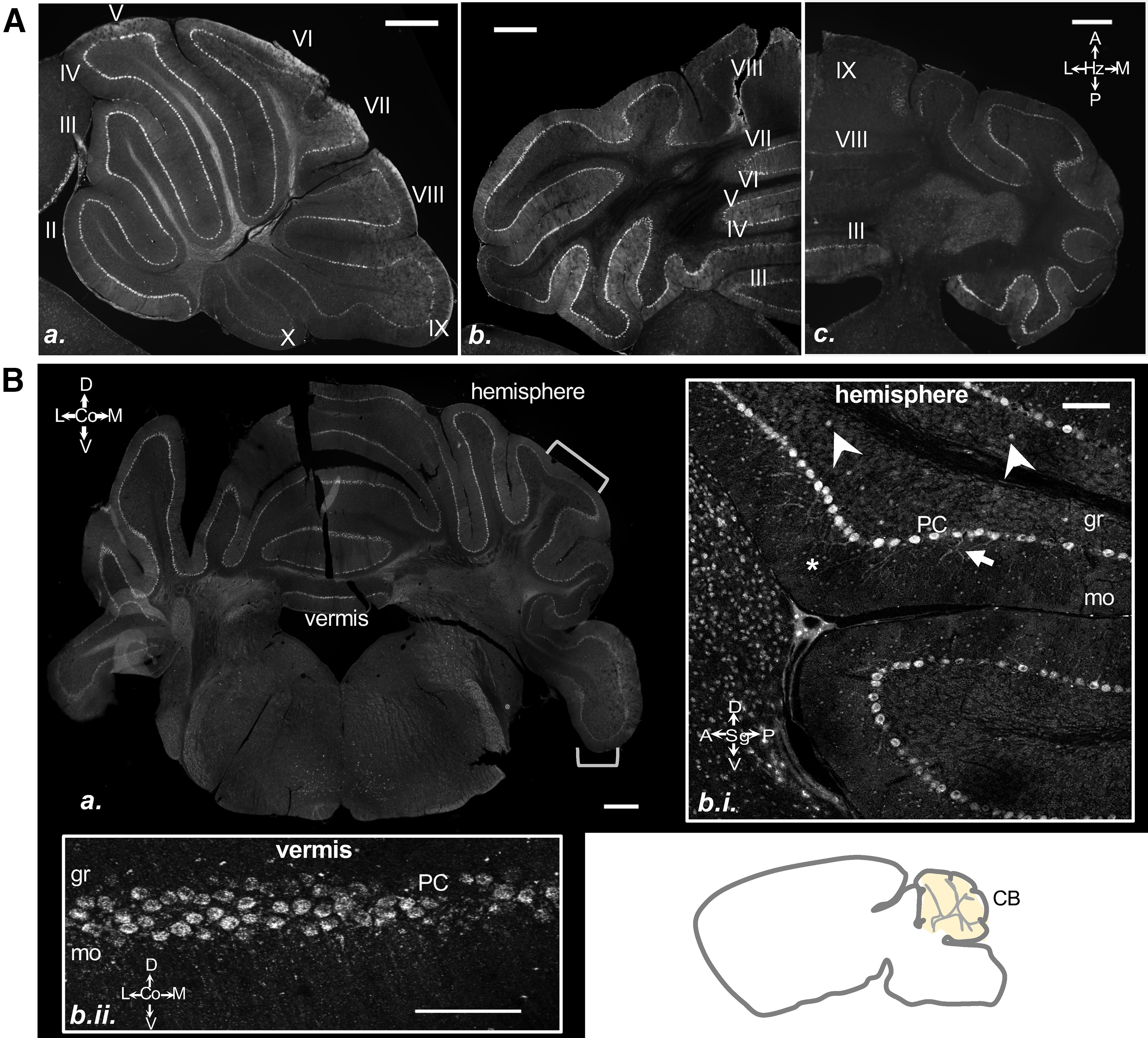
OPN3 expression in the cerebellum of the adult OPN3-mCh mouse brain. ***Aa***, OPN3-mCh expression in a sagittal view of the cerebellum (CB). Lobes are labeled with numerals. OPN3-mCh expression is strongest in Lobes II–VI and decreasingly lower in VII–X. ***Ab***, ***Ac***, Horizontal serial sections of the cerebellum with (***Ab***) more rostral than (***Ac***). OPN3-mCh expression decreases as the cerebellar lobes progress, with nearly negligible expression in Lobes VIII and IX. Scale bars: 500 μm. ***B***, A representative coronal section of the cerebellum with areas containing little to no OPN3-mCh expression (brackets) and areas with significant OPN3-mCh expression. Scale bar: 500 μm. ***Bbi***, Magnified sagittal image of the hemisphere showing OPN3-mCh in the granule layer (gr; arrowheads) and to a greater extent in the molecular layer (mo; asterisks). OPN3-mCh can be observed in the processes of the Purkinje cells (PC; arrow). Scale bar: 100 μm. ***Bbii***, Magnification of the Purkinje cell layer in the vermis of a coronal section. Scale bar: 100 μm.

Albeit consisting of only three studies, OPN3 expression in the cerebellum is the most widely studied of all OPN3-expressing regions (Blackshaw and Snyder, 1999; Lein et al., 2007; Nissilä et al., 2012). OPN3 mRNA was previously found in the Purkinje cell layer of both the vermis and hemispheres of the cerebellar cortex. Within the Purkinje layer, OPN3 mRNA follows a rostrocaudal gradient, having low to no expression within Purkinje cells in more caudal Lobes VIII–X. This gradient was only observed in mice greater than postnatal day 20 and was most prominent in the adult cerebellum (Blackshaw and Snyder, 1999). This gradient was not examined in the Nissilä et al. (2012) study, and it was unknown whether OPN3 protein followed the same pattern of expression. We examined the cerebellum from coronal, sagittal, and horizontal planes and confirmed that the gradient observed for OPN3 mRNA is conserved for the OPN3-mCh protein. In the adult OPN3-mCh mouse, we saw OPN3-mCh in the Purkinje cell layer decrease in expression in more caudal lobes (Fig. 10*A*). OPN3-mCh was present in the Purkinje layer of the vermis and both hemispheres, with strong mCh immunoreactivity in both the soma and proximal dendrites (Fig. 10*Bbi*). In addition, OPN3-mCh was also expressed in a subset of cells within the granule layer and in many neurons of the molecular layer (Fig. 10*B*; see also Figs. 5*Ac*, 6*Bc*). In the Blackshaw and Snyder (1999) and Lein et al. (2007) studies, OPN3 mRNA was observed to follow a radial striped pattern in the form of sharp delineated bands of expression across the vermis and hemispheres. We could not identify such marked banding patterns for OPN3-mCh protein expression, but, as in Lein et al. (2007), we observed that OPN3-mCh expression was not as intense at the outer edge of Lobe VI and in the paraflocculus (Fig. 10*Ba*).

#### Midbrain, pons, and medulla

**Figure 11.**
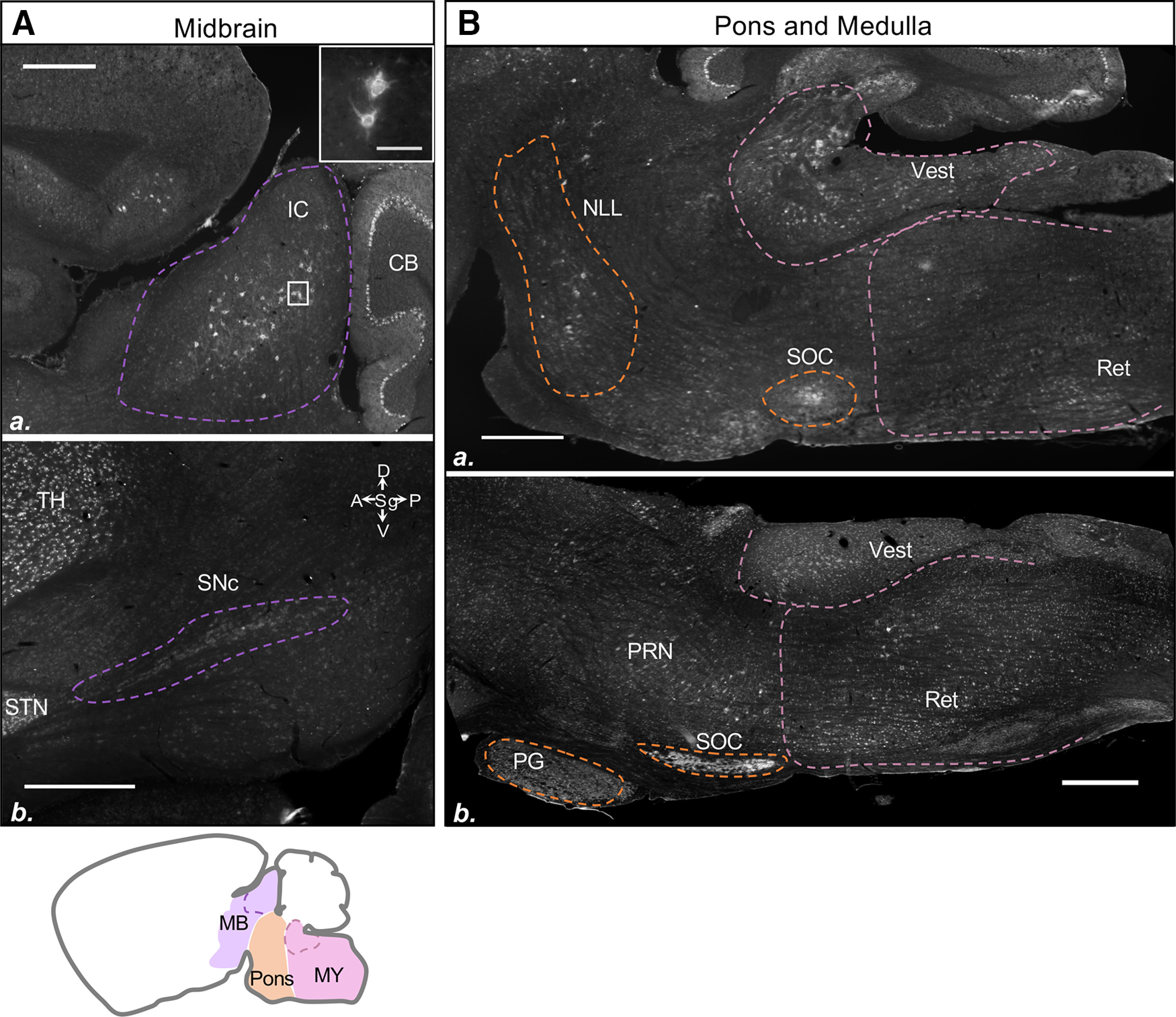
OPN3 expression in the midbrain, pons and medulla of the adult OPN3-mCh mouse brain. ***Aa***, Robust OPN3-mCh expression in the inferior colliculus (IC) by the cerebellum (CB). Scale bar: 500 μm. Inset, Higher magnification boxed region shows multidendritic branching of the OPN3-mCh+ cells. Scale bar: 100 μm. ***Ab***, OPN3-mCh shows distinct expression in the substantia nigra compacta (SNc) posterior to the subthalamic nucleus (STN). Scale bar: 500 μm. ***Ba***, Widefield image of the pons and medulla with regions of higher OPN3-mCh expression outlined. ***Bb***, A more medial section of the pons shows pontine reticular nucleus (PRN), pontine gray (PG), and superior olivary complex (SOC). Scale bars: 500 μm (***Ba***, ***Bb***). Vest: vestibular nuclei, Ret: reticular nuclei NLL: nucleus of the lateral lemniscus. Purple outline: midbrain (MB), orange outline: pons, pink outline: medulla (MY). Nissl counterstain can be found in Extended Data Figure 7-1.

The only previous investigation into OPN3 expression in the midbrain and hindbrain reported that embryonic day 18.5 mice have substantial levels of OPN3 mRNA in the dorsal pons (Blackshaw and Snyder, 1999). We sought to examine if this substantial expression persists in the adult OPN3-mCh mouse brain. We found that OPN3-mCh is only sparsely expressed in the adult midbrain and hindbrain. Within the midbrain, we discovered strikingly robust OPN3-mCh expression in the inferior colliculus among a subset of multidendritic cells (Fig. 11*Aa*). Less robust was OPN3-mCh expression in the substantia nigra compacta (Fig. 11*Ab*). In the pons, there was sparse expression in the nucleus of the lateral lemniscus and throughout the pontine reticular nuclei, moderate expression in the pontine gray, and high expression in the superior olivary complex (Fig. 11*B*). The medulla showed moderate OPN3-mCh expression dispersed throughout the vestibular and reticular nuclei, particularly in the medial vestibular nucleus directly below the cerebellum and the magnocellular reticular nucleus (Fig. 11*B*).

## Discussion

While the distribution of encephalic OPN3 mRNA has previously been examined (Blackshaw and Snyder, 1999; Halford et al., 2001; Dutra et al., 2020) and OPN3 expression for some brain regions has been studied using promoter-driven GFP reporter mice (GENSAT; Gong et al., 2003) and immunostaining (Nissilä et al., 2012; El Massri et al., 2018), our analysis of OPN3-mCh expression from coronal, sagittal, and horizontal planes across the adult OPN3-mCh brain has uncovered novel findings. We have first identified new regions in the brain in which OPN3 expression was previously unknown including: mitral cell layer, piriform area and nucleus of the lateral olfactory tract of the olfactory areas; CA regions and presubiculum of the HCF; posterior hypothalamic nucleus; inferior colliculus, and nucleus sagulum of the midbrain; and pontine gray and superior olivary complex in the pons (Extended Data Fig. 4–1, red text). We compared our OPN3-mCh expression with that of previous OPN3 mRNA and protein expression analyses, and in addition to our novel areas, our findings corroborate previous work (Extended Data Fig. 4-1). There were some instances of dissimilarity between our OPN3-mCh study and the OPN3 mRNA and reporter protein analyses, but these could be because of several factors (Extended Data Fig. 4-1, blue text). OPN3 is known to be developmentally regulated as its expression in the CTX, cerebellum, and pons differs from the embryo to adult (Blackshaw and Snyder, 1999). We focused our study only on the adult mouse which may have different expression profiles than neonates analyzed in previous reports. We may also see differences, especially from mRNA expression, due to differences in translational processing between mRNA and protein levels. This is explicit as little to no mRNA has been detected in the HCF [Allen Brain Atlas ISH Data (Lein et al., 2007)], yet we and others observe protein expression in the DG (Gong et al., 2003). We see the most differences between our OPN3-mCh expression mapping and that of the GENSAT OPN3-GFP reporter mouse. A major drawback to GFP reporters is the lack of endogenous degradation and turnover of the exogenous GFP, especially when OPN3 is developmentally regulated. In addition, BAC-based GFP reporters could be leaky and generate false positives. These potentially confounding factors are eliminated in our OPN3-mCh model in which the reporter fluorophore is fused to OPN3. The OPN3-mCh mouse also gives the ability to discern developmental regulation because OPN3-mCh is presumably degraded as the endogenous protein would be.

Additionally, we discovered that OPN3 is expressed not only in neurons but also in astrocytes within the HCF. This is the first indication of OPN3 in astrocytes. We are also the first to characterize horizontal sections which, in addition to coronal and sagittal sections, allowed us to uncover the GABAergic nature of many OPN3 cells, determine the non-ChAT nature of OPN3 cells in the striatum, and reveal of OPN3 subcellular localization in the soma and some cell terminals.

We find that OPN3 is expressed throughout the adult brain, but it displays differential distribution (Figs. 2-4). One unifying factor among all regions of OPN3-mCh expression is the presence of cytoplasmic OPN3-mCh predominantly in the soma with little to no immunostaining in processes. This is consistent with previously reported cellular distribution of OPN3 protein, albeit the immunostaining resolution is poor in the reported images (Nissilä et al., 2012). In some instances, however, especially larger cells with thicker dendrites, we do observe OPN3-mCh in regions most proximal to the soma; as processes extend radially from the cell, OPN3-mCh expression fades [see CTX pyramidal neurons (Fig. 7*A*); Purkinje cells (Fig. 10*B*); inferior colliculus (Fig. 11*A*)]. This could indicate that OPN3-mCh is expressed only at low levels in processes and with smaller cells, the combination of thinner processes and low OPN3-mCh expression makes OPN3 detection difficult. It is unlikely that the mCh tag used for the OPN3-mCh mice would adversely affect or limit endogenous OPN3 distribution to cell processes. This was a major concern of ours even before generating the OPN3-mCh mouse model and we took precautions to the best of our ability to ensure that OPN3-mCh maintained endogenous localization. In our previous publication, we identified that OPN3 subcellular localization in melanocytes and other heterologous cell types is at the plasma membrane, as expected of a GPCR, but also in internal vesicles within the cytoplasm; OPN3-mCh significantly colocalized with endogenous OPN3 (using the now discontinued OPN3 antibody) in primary human melanocytes (Ozdeslik et al., 2019). In addition to retaining its subcellular localization, we found that OPN3-mCh retained its regulatory activity in melanocytes, suggesting that OPN3-mCh is functional despite fusion to a fluorescent protein (Ozdeslik et al., 2019). Although there is a possibility that OPN3 processing may lead to altered subcellular localization of OPN3-mCh, we believe this is unlikely, as tagging other GPCRs at the direct C terminus with fluorescent proteins and using similar knock-in methods did not alter subcellular localization (as in the MC4R-GFP mouse; Siljee et al., 2018). We can also confidently exclude the possibility that mCh is cleaved after protein processing as mCh is a small (∼27 kDa), soluble protein with cellular localization very different from that of the larger OPN3-mCh (∼49 kDa), with seven transmembrane domains typical of all GPCRs; based on its size and topology, mCh localizes within the somas and terminals of the cells (Chen et al., 2013), whereas we consistently observe a punctate or granular appearance to the OPN3-mCh staining (Fig. 5, magnified images as examples), consistent with a membrane protein expressed at the plasma membrane and in membrane-bound intracellular compartments.

Alternatively, particularly in the HCF where OPN3-mCh appeared to be expressed within somas and minimally in dendrites in the DG granule and CA1/2/3 pyramidal cell layers (Figs. 5, 6*Bb*, 8), it is possible that some of the apparently somatic staining is actually staining of axon terminals that innervate these layers. The pattern of punctate OPN3-mCh staining, especially around the pyramidal cell and the granule layers, could be consistent with high OPN3-mCh expression in the axon terminals of GABAergic interneurons innervating the pyramidal and granule cells (Freund and Buzsáki, 1998) and not solely OPN3-mCh expression in the somas of the pyramidal and granule cells. The present work did not attempt to discriminate between somatic labeling versus innervations of cells by axon terminals, but this may be of interest for future studies.

Taken as a whole, OPN3-mCh expression patterns broadly follow circuitry related to sensorimotor pathways. OPN3-mCh has notably high expression in the olfactory areas including the AOB, piriform area, olfactory tubercle and many layers of the main olfactory bulb. It is well known that cortico-thalamo-cortical pathways are essential in the processing of most sensations, among a myriad of other functions (Guillery and Murray Sherman, 2002). OPN3-mCh is present in the somatosensory areas of the CTX which form connections with the hub of sensory circuitry, the thalamus, the region where OPN3-mCh is most highly expressed. The ventral posterior nuclei of the thalamus where OPN3-mCh expression is prominent, aids in processing somatosensory input, among other functions (Pierret et al., 2000; Bureau et al., 2006; Wimmer et al., 2010; Ohno et al., 2012; Oh et al., 2014). OPN3 is also expressed in the mouse retina (Nissilä et al., 2012; Buhr et al., 2015), possibly explaining OPN3-mCh expression in the LGd of the thalamus, which is the terminal region of most retinal ganglion cells (Szentagothai, 1963). OPN3-mCh is furthermore expressed in many regions essential for the relay and processing of auditory information: auditory cortex, medial geniculate complex of the thalamus, inferior colliculus and superior olivary complex of the midbrain and hindbrain, respectively (Oh et al., 2014; Blazquez Freches et al., 2018). We also found OPN3-mCh in the motor nuclei of the thalamus, the motor area of the CTX, Purkinje cells, and other nuclei of the cerebellum and in ventral pontine nuclei.

In addition to possibly being involved in larger, systems-level functions like sensation and motor control, it is likely that OPN3 performs region-specific functions in a variety of different cell types (neurons and GFAP+ cells) that may, or may not, work together toward a unifying role. For example, we have shown that OPN3-mCh is expressed in GABAergic Purkinje cells of the cerebellum and GABAergic cells in the DG (Fig. 6), yet it is also expressed in the excitatory pyramidal and granule cells of the HCF and excitatory cells of the thalamus (Figs. 8, 9). It is evident that OPN3 does not simply follow a neuronal phenotype-specific pattern but is regulated in a more complex region-specific manner and even within the same brain region by different inputs and microdomains. For instance, the most intriguing and unanticipated expression of OPN3-mCh was in the HCF. In the HCF, OPN3-mCh is surprisingly abundant given that previous mRNA reports (Allen Brain Atlas ISH Data) have indicated no expression in this area (Lein et al., 2007). As mentioned, differences in mRNA regulation and translation into protein may account for the differences. There is one other report from the GENSAT database of OPN3 being highly expressed in the granule layer of the DG, but no further studies examined HCF protein expression (Gong et al., 2003). This is the only region in which we readily observe star-like, GFAP+ astrocytic OPN3-mCh immunoreactivity in addition to its presence in neurons. Although astrocytes are present throughout the brain, they, like neurons, have different morphotypes, respond to different stimuli, and have different functions (for review, see Miller, 2018). Even within the HCF itself, astrocytes exhibit differences between the CA layers and the DG layers (Matyash and Kettenmann, 2010; Jinno, 2011). Indeed, it has been shown using DREADDs and endogenous receptors that astrocytes of the hippocampal stratum radiatum (as opposed to the striatum) have differential Gi-coupled GPCR signaling that may result from variation in gene expression (Chai et al., 2017). Because OPN3 in a variety of species, including human, has been shown to couple to the G-protein α-subunit Gi (Koyanagi et al., 2013; Sugihara et al., 2016; Ozdeslik et al., 2019), OPN3 could exert specific Gi-based signaling or regulation specific to the HCF, explaining its strong expression in astrocytes in the HCF versus other regions.

OPN3, as a member of the opsin family of light-receptive GPCRs, may also participate in light-evoked processes in the brain. OPN3 homologs in mosquito, pufferfish, zebrafish, and chicken absorb blue light (Koyanagi et al., 2013; Sugihara et al., 2016). Human OPN3, however, does not absorb visible light by UV-visible spectroscopy (Ozdeslik et al., 2019), despite several studies indicating OPN3-meditated blue light responses in mammals (Kato et al., 2016; Buscone et al., 2017; Barreto Ortiz et al., 2018; Regazzetti et al., 2018; Yoshimoto et al., 2018; Castellano-Pellicena et al., 2019; Nayak et al., 2020; Sato et al., 2020). In lower vertebrates, photosensitive areas in the diencephalon contain many light-responsive pigments and opsins that directly mediate physiological reactions like color change and motor responses independent of the eyes or pineal glands (Van Veen et al., 1976; Tabata, 1982). It has been demonstrated in a variety of mammalian species from rodents to humans that visible and near infrared light is capable of penetrating through the skull into the brain, albeit not very far (Van Brunt et al., 1964; Viggiani et al., 1970; Hartwig and van Veen, 1979; Kobat et al., 2011). Most of the visible light is scattered by bone and tissue, but in smaller animals like rodents, a small percentage of light (red > blue > green; Viggiani et al., 1970) can penetrate into the brain, even to diencephalic regions like the hypothalamus (Van Brunt et al., 1964).

If OPN3 were to act as a photoreceptor, it would require a chromophore, most likely 11-*cis* retinal, for activation; the brain has a supply of retinoids and intriguingly, RPE65, the enzyme that catalyzes the isomerization of all *trans* retinyl ester to 11-*cis* retinol was shown to be expressed in cells lining the third ventricle (Shearer et al., 2010; Ransom et al., 2014), located just ventral to the PVH. We have found OPN3-mCh expression explicitly in the PVH of our knock-in mouse. It is therefore conceivable that OPN3, even in deep diencephalic regions of the mouse brain, has the resources available to act in a photosensitive manner.

Despite the widespread expression of OPN3 shown in this study, the OPN3 knock-out mouse shows no obvious phenotype. Although we can speculate on the potential brain functions of OPN3 based on its expression patterns, the functional roles of OPN3 in the brain remain elusive. It is likely that the OPN3 knock-out mouse has a yet unidentified neurologic phenotype that does not manifest in the conditions or life span to which we house our mice. Indeed, the knock-out mouse was first generated years ago (Buhr et al., 2015), but it has just recently been discovered that only when perturbing normal living and physiological conditions does the OPN3 knock-out mouse exhibit diet-induced obesity and insulin resistance because of dysregulation of metabolic processes in brown adipocytes (Sato et al., 2020) and deficiency in thermogenesis under cold shock because of dysregulation of lipolysis in adipose tissue (Nayak et al., 2020). Interestingly, none of these phenotypes have demonstrated correlations to OPN3 expression in the brain.

While the functional roles of OPN3 cannot be determined solely on its expression pattern, we have made a large step in understanding potential roles for OPN3 in the brain. With the generation of the OPN3-mCh mouse, we have created a novel tool that will further the investigation of OPN3 and will allow for the complete characterization of OPN3 not only in different areas of the brain, but also in all tissues within the mouse. Our model allows the identification of subcellular localization of OPN3 across the entire organism, examination of protein interactions and colocalization without reliance on OPN3 antibodies, and analysis of developmental regulation of the receptor. The generation of the OPN3-mCh mouse model will ignite and facilitate new research into OPN3 function, an area that has lain stagnant for several decades largely because of a lack of reliable and specific identification methods for OPN3 expression.

## Synthesis

Reviewing Editor: Kirill Martemyanov, The Scripps Research Institute Decisions are customarily a result of the Reviewing Editor and the peer reviewers coming together and discussing their recommendations until a consensus is reached. When revisions are invited, a fact-based synthesis statement explaining their decision and outlining what is needed to prepare a revision will be listed below. The following reviewer(s) agreed to reveal their identity: Seth Blackshaw.

Upon discussion the reviewers agreed that the study is technically sound and found the data of adequate quality. There was also a sense that the mouse model generated could be a useful reagent for the field. However, the reviewers also pointed out that the study does not really break new ground as most of the results confirm earlier findings. It was felt that the revision needs to explicitly address the significance issues and point out the ways in which their reagent could be used to advance further studies on Opn3 and thoroughly discuss what new information this report provides relative to what is already known. In addition, addressing the specific issues brought up by the reviewers will be needed during the revision. Please refer to comments below.

Reviewer #1

This manuscript reports the generation and characterization of an in-frame C-terminal Opn3-tdTomato knock-in mouse line. The study focuses on describing the expression pattern of Opn3-tdTom in adult brain, which is essentially identical to the previously reported distribution of Opn3 mRNA. The manuscript could be improved by addressing several outstanding issues, which are listed below:

Major points:

1. The cellular and regional expression patterns reported here essentially identical to the mRNA expression patterns reported in Blackshaw and Snyder, 1999. A more detailed dataset has been produced by the Allen Brain Atlas, and is described further in Lein, et al. 2007. Similarities and differences between the mRNA and Opn3-tdTom expression patterns should be explicitly listed in a modified Figure 3.

2. The authors repeatedly refer to available anti-Opn3 antibodies as being ineffective. What is the evidence for this? Data showing that immunoreactivity is preserved in Opn3 null mice would be sufficient.

3. The finding that Opn3-tdTom fluorescence is largely restricted to the cell soma is interesting, but potentially confounded by the presence of the C-terminal fusion, which may disrupt subcellular targeting in neurons. In the absence of any direct experimental data addressing this, published studies should be reviewed to determine the likelihood that this might pose a problem.

4. The authors state that homozygous mice were used for the study. Were any differences in expression pattern or subcellular localization of tdTom detected between homozygotes and heterozygotes?

5. The key novel findings of this study should be explicitly summarized in the Discussion. Specifically, these include the finding that fluorescence is largely confined to the cell soma, the identification of astrocyte-specific expression in the hippocampus, and the analysis of expression in Gad67-positive neurons in multiple brain regions.

Minor comments:

Pg. 2: Opn3 was characterized using both radioactive and non-radioactive in situ hybridization in Blackshaw and Snyder, 1999.

Pg. 3: “This study presents the first step in understanding the elusive expression and function of OPN3 in the brain”. This overstates things a bit, considering that the mRNA expression pattern has been described in great detail, and is essentially identical to the Opn3-tdTom expression here.

Pg. 11: Opn3 expression in AOB has been previously shown by the Allen Brain Atlas.

Pg. 18: Replace “most often” with “most likely”.

Reviewer #2

This manuscript provides a comprehensive characterization of Opn3 protein expression in the mouse brain using a new mouse line where Opn3 has been tagged with the mCherry protein. Overall, this paper is comprehensive and will provide an important resource for those studying the function of this opsin, the function of which is still not well understood. This paper also describes an important new genetic tool for the study of Opn3 function and expression in mice.

Concerns:

1. My major concern lies with the fact that there was only minimal validation that this mouse line does indeed represent Opn3 expression. The authors only compare the Opn3LacZ and Opn3-mCh lines in the Purkinje cells of the cerebellum, but nowhere else in the brain. How do we know these newly identified areas truly contain Opn3? As the authors state that the antibodies to Opn3 are problematic, that leaves us to assume that the new mouse line these authors describe is indeed valid based on a comparison in expression of one cell type to that of another mouse line that was also substandard enough to warrant generation of a new tool to visualize Opn3 expression.

The authors touch on other studies looking at the distribution of Opn3 expression, but mostly in the context of what has been newly identified. At a minimum, it would be helpful to more explicitly summarize the comparisons between their findings and those of others in the discussion in a standalone section or paragraph. Are there areas labeled by others (in mice) that do not show up in the Opn3-mCh line or does this line encompass everything we already knew and some additional areas? Beyond that, it would be most helpful if the authors could provide additional experimental corroboration in some cases. For example, the authors identify potential Opn3 expression in astrocytes of the HCF, and this would benefit from some additional corroboration of Opn3 expression since it has not been identified using any other methods. However, I do not think it is strictly necessary (given the difficulty for labs in completing experiments currently) as long as they are very clear about how the labeling compares to the multiple other approaches in the discussion and where and how the labeling differs. This will make the tool even more useful to researchers interested in studying Opn3.

2. Figure 3 shows a nice summary of Opn3 expression across coronal sections in the brain. It would be helpful to show actual coronal sections from the mouse line itself for comparison here. Presumably the authors have this already, and it would be helpful to include so that the reader can compare the schematic to the raw data.
