# Supplementary figures and images for: Endogenous Opsin 3 (OPN3) Protein Expression in the Adult Brain Using a Novel OPN3-mCherry Knock-In Mouse Model
Source: eNeuro. 2020 Sep 2;7(5):ENEURO.0107-20.2020. doi: 10.1523/ENEURO.0107-20.2020 (PMC7477952; doi:10.1523/ENEURO.0107-20.2020)

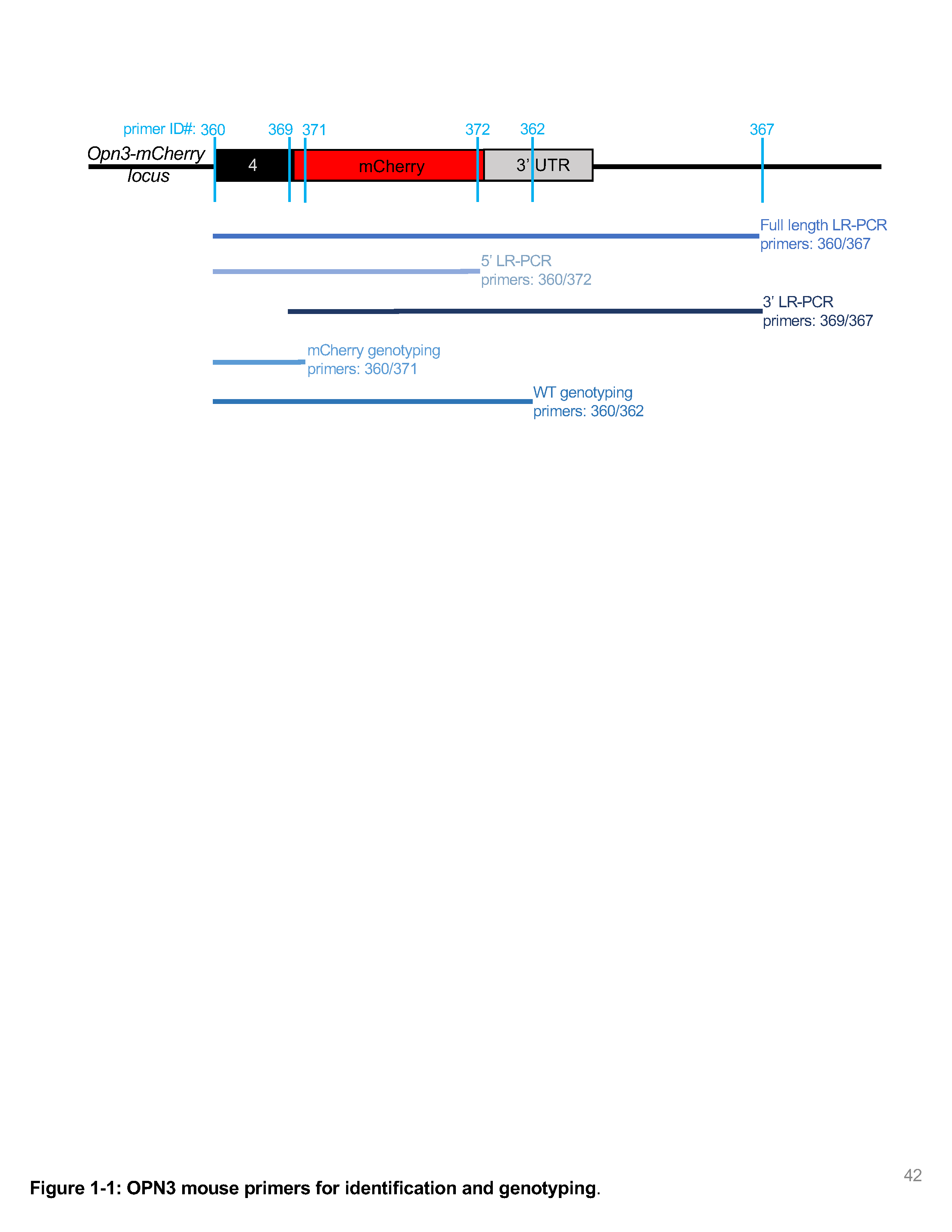

Supplement: Extended Data Figure 1-1 — OPN3 mouse primers for identification and genotyping. Primers used to verify mCherry insertion and for subsequent genotyping. Primer names and pairs correspond to Materials and Methods, Primers and sgRNA sequences. LR-PCR: long-range PCR, SR-PCR: short-range PCR Download Figure 1-1, TIF file. [file enu-eN-MNT-0107-20-s03.tif]

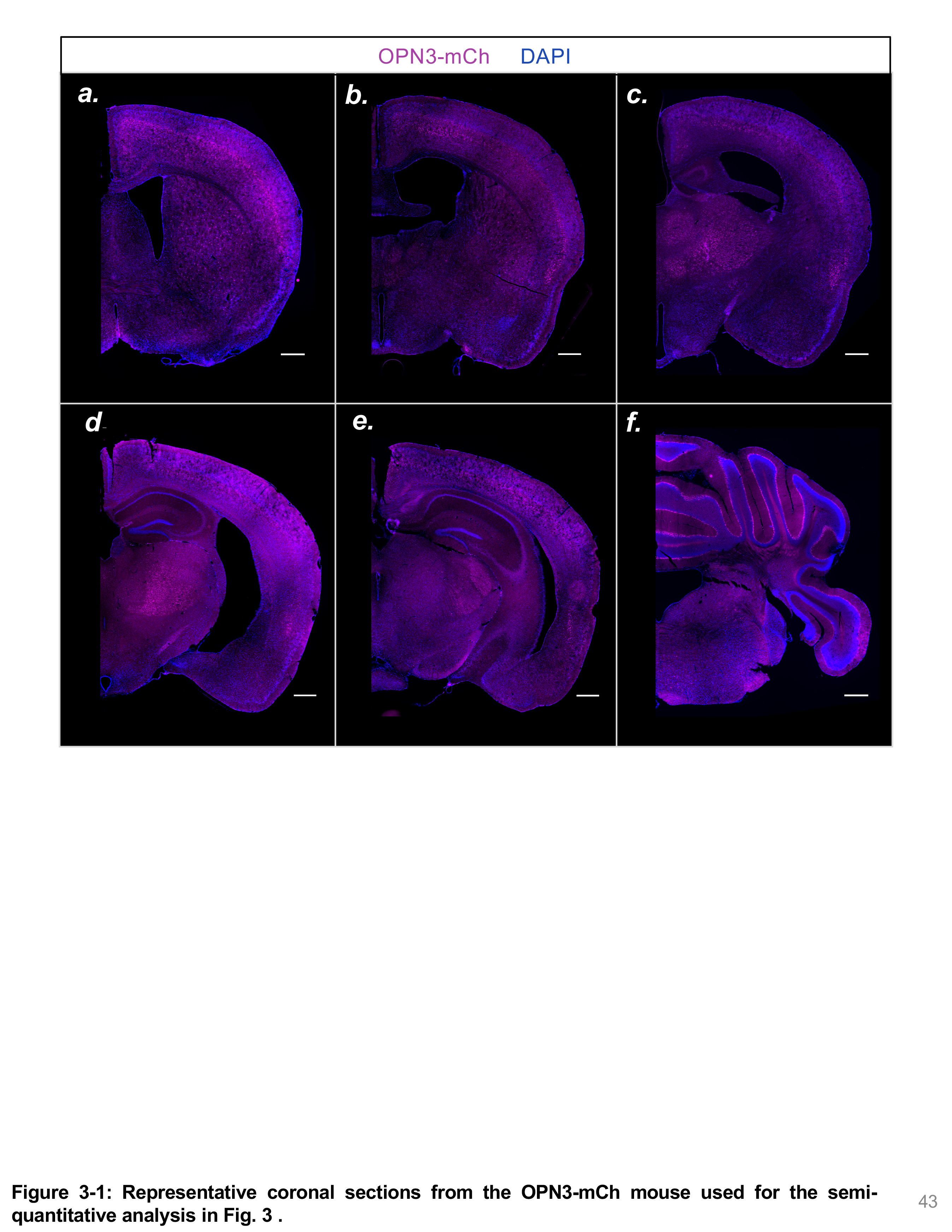

Supplement: Extended Data Figure 3-1 — Representative coronal sections from the OPN3-mCh mouse used for the semi-quantitative analysis in Figure 3. Fluorescent images of coronal sections from homozygous OPN3-mCh mice, roughly corresponding to the planes represented in Figure 3. OPN3-mCh is in magenta and DAPI counterstain is in blue. All scale bars: 500 μm. Download Figure 3-1, TIF file. [file enu-eN-MNT-0107-20-s04.tif]

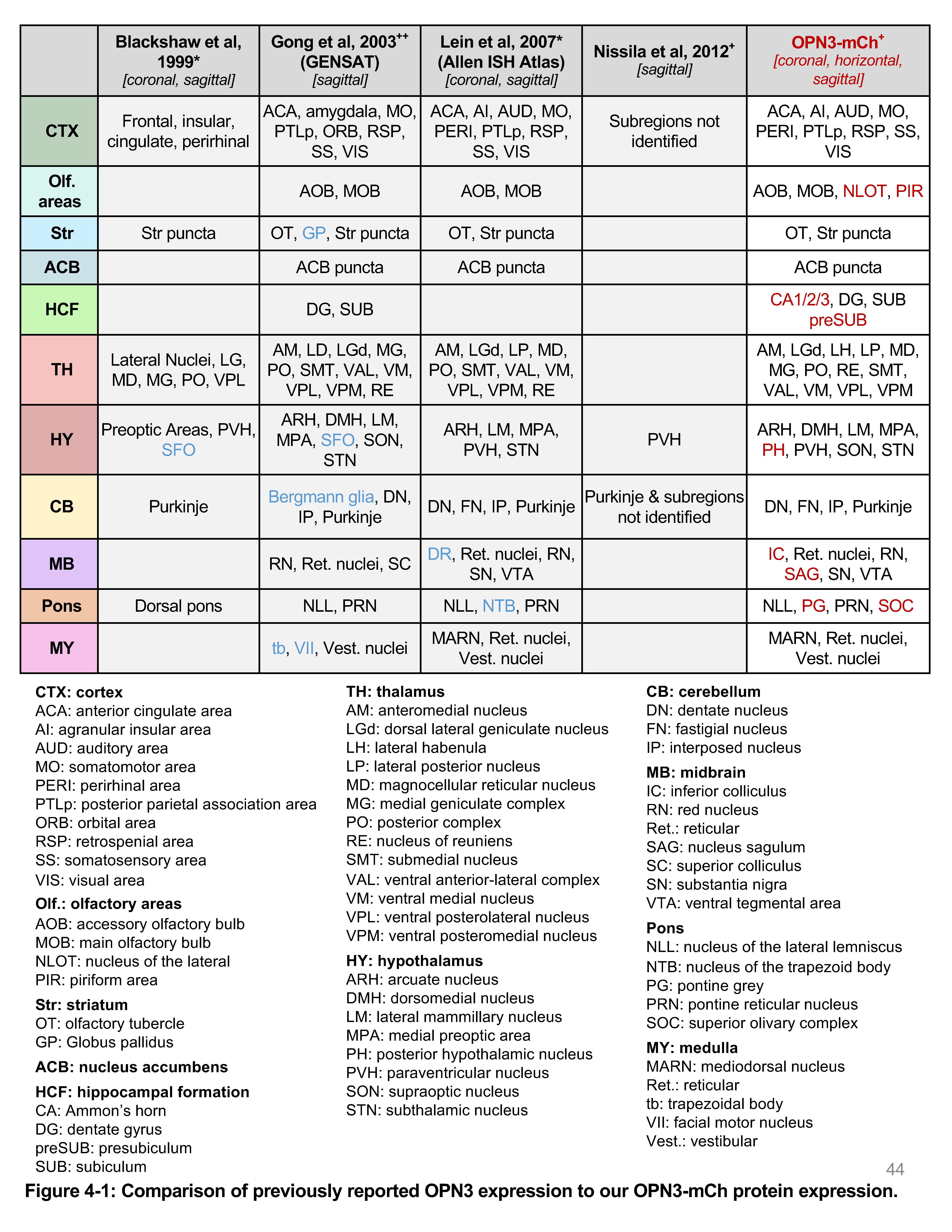

Supplement: Extended Data Figure 4-1 — Comparison of previously reported OPN3 expression to our OPN3-mCh protein expression. Previously published or publicly accessible OPN3 mRNA or protein expression in the mouse brain as compared to our OPN3-mCh expression data by structure. In red are newly identified areas of OPN3 expression revealed by the OPN3-mCh mouse. In blue are areas of OPN3 expression found previously but not detected by the current OPN3-mCh analysis. Allen Brain Atlas ISH Data and GENSAT subregions were interpreted from publicly available brain sections from C57BL/6J (P56) and Tg(Opn3-EGFP)JY3Gsat/Mmucd (P7, adult) mice, respectively. *, OPN3 mRNA; +, endogenous OPN3 protein; ++, reporter for OPN3. Download Figure 4-1, TIF file. [file enu-eN-MNT-0107-20-s05.tif]

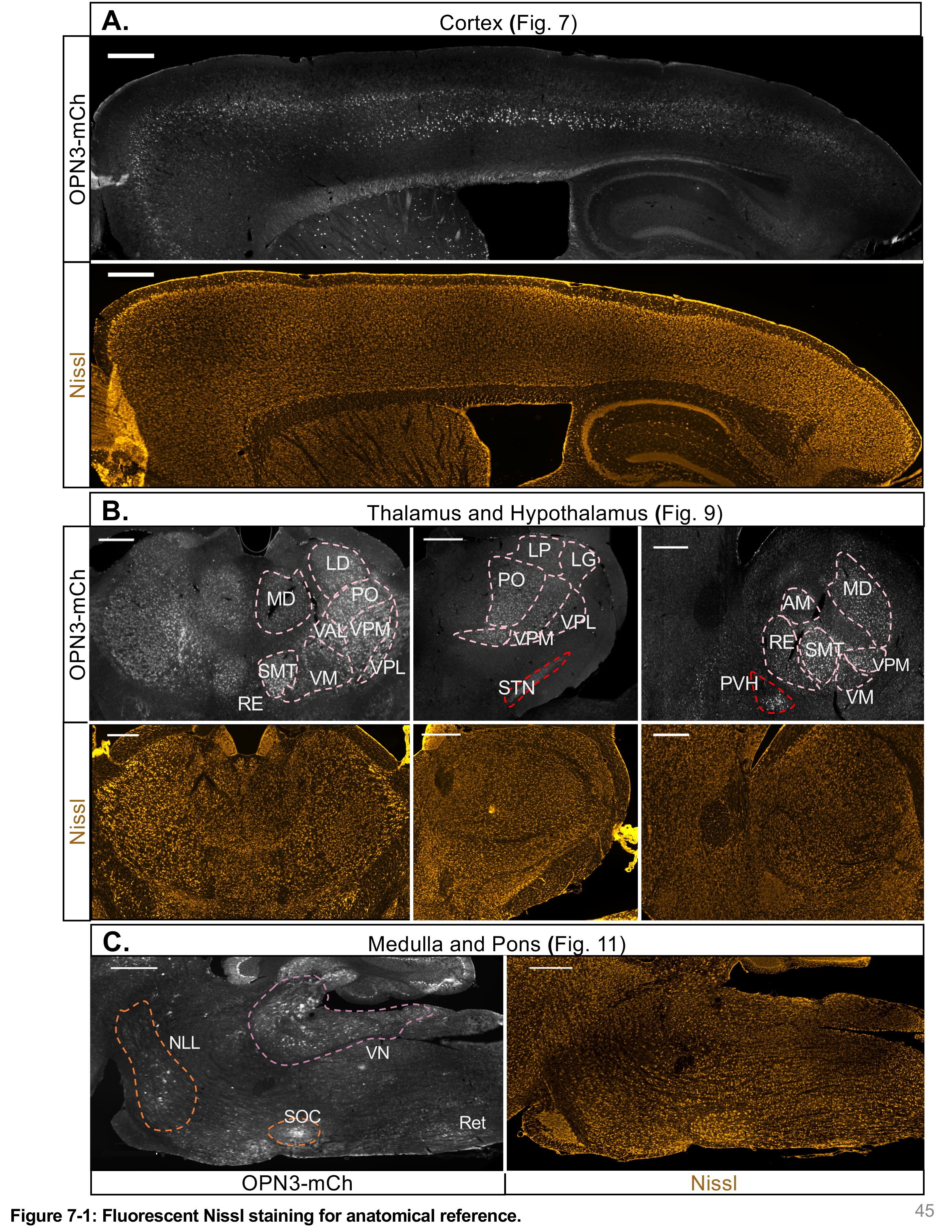

Supplement: Extended Data Figure 7-1 — Fluorescent Nissl staining for anatomical reference. Fluorescent Nissl stain of sections immediately preceding or anteceding sections shown in Figures 7, 9, 11. All scale bars: 500 μm. Download Figure 7-1, TIF file. [file enu-eN-MNT-0107-20-s06.tif]
